# From Percentiles to Distributions: Nationwide ZCTA-Level Modeling of Indoor Radon Exposure across the Contiguous United States, 2000–2021

**DOI:** 10.1021/acs.est.6c01333

**Published:** 2026-08-31

**Authors:** Teng Yang, Longxiang Li

**Affiliations:** Gangarosa Department of Environmental Health, Rollins School of Public Health, 1371 Emory University, Atlanta, Georgia 30322, United States

**Keywords:** indoor radon, exposure assessment, distributional characteristics, small areas, geological model

## Abstract

Radon exposure is the leading cause of lung cancer among nonsmokers, posing a significant public health risk. Despite its known dangers, population-wide exposure assessments of indoor radon have been limited by the reliance on area-based summaries, such as average and median concentrations, which ignore the distributional characteristics of small areas. This study developed a national-scale modeling framework to estimate ZIP Code Tabulation Area (ZCTA)-level distributions of indoor radon concentrations across the contiguous United States from 2000 to 2021. We utilized over six million short-term radon measurements, which were aggregated at the ZCTA level to predict percentiles. By further modeling within-ZCTA radon exposure distributions, we improved the quantification of exposure heterogeneity and were able to estimate the proportion and number of residents exceeding policy-relevant exposure thresholds more accurately. The results reveal that approximately half of U.S. ZCTAs have at least one in five households exposed to radon concentrations exceeding the Environmental Protection Agency’s action level (148 Bq/m^3^), with high-exposure areas predominantly located in the Midwest. These findings provide crucial data to help residents recognize their potential radon risk, urge policymakers to design cost-effective interventions, and offer health researchers an essential database for incorporating within-ZCTA radon exposure distributions into health risk assessments.

## Graphical abstract

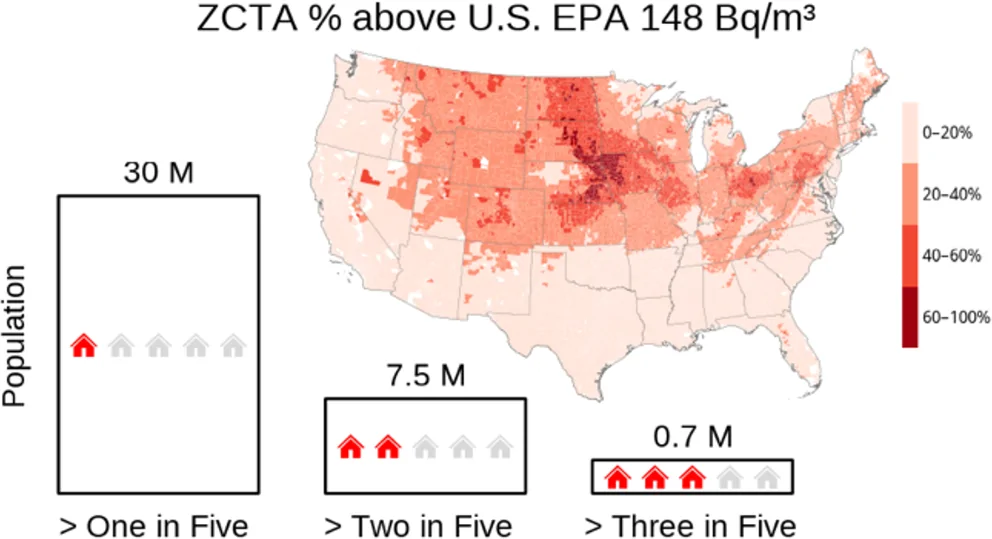

## Introduction

Radon is an invisible, odorless, and radioactive gas that originates from the natural decay of uranium in soils and rocks and can accumulate indoors, particularly in poorly ventilated buildings. Indoor radon exposure is an often-underestimated environmental hazard and the leading cause of lung cancer among nonsmokers. In the United States (U.S.), radon is estimated to account for approximately 21,000 lung cancer deaths annually, including about 2,900 deaths among individuals who have never smoked.^1^ Emerging evidence further suggests associations between radon exposure and other adverse health outcomes.^2–6^ To reduce radon-related health risks, the U.S. Environmental Protection Agency (EPA) recommends mitigating indoor radon levels to below 4 pCi/L (148 Bq/m^3^).^7^ Despite this recommendation, awareness of radon exposure remains limited^8^ and indoor radon is not subject to systematic monitoring. Unlike ambient pollutants that are routinely monitored and regulated, indoor radon data are not collected through a systematic, national monitoring network. Instead, many available measurements are generated through property transactions, surveys, or voluntary testing initiated by residents. This situation highlights a fundamental tension between the strong public health need for high-quality, population-representative radon exposure data and the fragmented, heterogeneous nature of existing radon measurements.

Radon maps have therefore been widely used to describe spatial patterns of indoor radon exposure and to identify areas with elevated radon levels. Most existing studies have focused on estimating the prevailing radon concentrations within predefined spatial units.^9–14^ For example, a Bayesian mixed-effects regression model developed by Lawrence Berkeley National Laboratory predicted county-level geometric mean radon concentration,^9,10^ and our prior work using random forest models estimated monthly average radon levels at the community level across the contiguous U.S.^11^ However, area-level summary radon concentrations provide limited support for population-wide risk communication. Given the highly right-skewed distribution of indoor radon concentrations, central tendency measures alone cannot characterize the full within-area distribution or directly quantify the proportion of homes or residents exceeding policy-relevant thresholds. Meanwhile, population-based epidemiological studies, especially those with individual-level demographic information, generally rely on area-based radon estimates as proxies for personal exposure.^2,3,5,15^ While such approaches are practical, they implicitly assume the homogeneity of radon exposure within spatial units and fail to capture within-area distributional characteristics, including the spread and upper tail of indoor radon concentrations. Such simplification may result in exposure misclassification,^16^ especially for individuals experiencing high-end exposures that are more relevant to health risks.

To date, relatively little is known about the within-area distributional characteristics of radon exposure at the population level, particularly in the U.S. Recent studies have begun to move beyond area-level summaries by adopting approaches that aim to characterize exposure distribution.^17,18^ Some studies estimate within-area dispersion by aggregating observed radon measurements. For example, the European Indoor Radon Map (EIRM) reports annual standard deviations of ground-level indoor radon concentrations for 10 km × 10 km grid cells.^17^ However, these estimates are highly sensitive to sampling density and remain unreliable across large portions of the study area.^19^ A recent German study advanced indoor radon mapping by using a probabilistic aggregation framework. Petermann et al. trained a quantile regression forest to predict floor-specific indoor radon quantiles for each residential building in Germany, fitting three-parameter log-normal distributions to the predicted quantiles, and applying population-weighted Monte Carlo sampling to estimate administrative-level distributions and threshold exceedance probabilities.^18^ While representing an important methodological advance, such aggregation is challenging to apply directly in the U.S. setting because it depends on detailed information or assumptions about building characteristics and floor-level population distributions that are difficult to observe comprehensively at large spatial scales.

Therefore, in this study, we developed a national-scale modeling framework to estimate ZIP Code Tabulation Area (ZCTA)-level distributions of indoor radon exposure across the U.S. from 2000 to 2021. By jointly predicting multiple percentiles of radon concentrations and constructing full exposure distributions at the ZCTA level, our approach enables direct quantification of within-area heterogeneity and the identification of high-exposure subpopulations that are likely obscured by area-level summary estimates. This distributional approach advances radon exposure assessment beyond prevailing methods and offers a more informative basis for population-level risk communication.

## Materials and Methods

### Radon Measurements from National Database

The national radon measurements used in this study closely follow our prior work.^11^ The data set includes over six million short-term residential tests collected across 35 U.S. states during property transactions. For addresses with multiple measurements, only the first measurement was retained. Measurements below the detector-specific lower detection limits (LDLs) were imputed using a maximum likelihood-based left-censored approach, as detailed in Text S1.^20^ At the national scale, radon concentrations showed a strongly right-skewed distribution,^11^ whereas their log-transformed values approximated a normal distribution (Figure S1). The distributions by regions generally followed similar log-normal patterns, with meanlog values ranging from 4.26 to 4.67 and sdlog values ranging from 1.12 to 1.19, although central tendency and variability differed across regions (Figure S2).

We subsequently grouped individual measurements by 5-digit ZCTA and the calendar month of measurement. To characterize the within-ZCTA distribution and parametrize the log-normal distribution, we calculated the 10th, 25th, 50th, 75th, and 90th percentiles for each ZCTA–month combination that included at least five residential measurements. Empirical percentiles were calculated by linear interpolation between adjacent ordered observations.^21^ We also quantified key measurement attributes, including the proportions of tests conducted in basements versus above-ground floors and the distribution of the four detector types. Furthermore, we expanded the training data set by iteratively resampling the radon observations with replacement for ZCTA months with at least 9 measurements. Finally, we applied a logarithmic transformation to the monthly ZCTA-level observations to address their right-skewed distribution. After completing all data-processing steps, we obtained a total of 339,671 monthly ZCTA-level radon observations, of which 39.4% were derived from original measurements and 60.6% were generated through resampling. Additional methodological details are provided in our previous work.^11^

### Radon Predictors

We assembled 189 predictors to estimate the monthly ZCTA-level radon concentrations. These predictors were grouped into six major categories: detector-related, geological, architectural, socioeconomic, meteorological, and policy factors (Table S1). In addition, we included the calendar year to account for long-term trends and the month of the year to account for seasonality that has not been fully captured by meteorological factors. We also included the longitude and latitude to account for the spatial trend that was not fully captured by geological factors. Detailed information on the radon predictors is provided in Text S2 and has been described previously.^11^

### Data Analysis

We predicted monthly ZCTA-level radon percentiles using a random forest model, an ensemble learning approach that aggregates multiple decision trees trained on bootstrapped samples.^22^ Our previous national radon modeling study showed that random forest outperformed other approaches such as gradient boosting, neural networks, and ensemble models.^11^ For each percentile, we developed separate prediction models using the same sets of detector-related, policy-related, spatial, and temporal predictors, as well as percentile-specific geological, architectural, socioeconomic, and meteorological predictors. All predictors were processed through centering and scaling. Training weights were incorporated into random forest models to account for both state-level sampling representation and variation in the number of measurements across ZCTA months. During model development, we evaluated multiple hyperparameter combinations and selected a common near-optimal setting for all percentile-specific models to ensure consistency and improve computational efficiency. The selected hyperparameter setting used 300 trees, a maximum tree depth of 40, an mtry value of 60, a minimum node size of 1, and an extremely randomized trees splitting rule. We assessed variable importance at the category level to reduce potential dilution of importance across correlated predictors within the same class and to improve the interpretability of relative predictor contributions in our prediction model, as detailed in Text S3.

We fit a classic random forest model using the “ranger” package (version 0.17.0)^23^ in R (version 4.2.2). All analyses were conducted on the High-Performance Computing (HPC) cluster, which is supported by the Rollins School of Public Health at Emory University. The cluster includes 25 general-use compute nodes, 24 of which have 32 compute cores and at least 196 GB of RAM each.

### Model Evaluation

We evaluated model performance using repeated 10-fold cross-validation (10-fold CV). The full training data set was randomly split into ten mutually exclusive folds. In each iteration, nine folds were used to fit the random forest model and the remaining fold was used for validation. This process was repeated three times to generate stable estimates of the predictive accuracy. Model performance was summarized by using the mean absolute error (MAE) between the log-transformed predicted and observed radon percentiles. We further evaluated model performance using the mean relative absolute error (MRAE), a scale-independent metric that enables comparison across models predicting different percentiles. We stratified the comparisons by sample size and computed size-specific metrics across five percentiles of the radon distribution.

Based on our prior work, ZCTA-level observation (*C*_o_) is a reliable estimation of actual concentration when derived from a large sample.^11,24^ Therefore, we evaluated prediction (*C*_p_) against *C*_o_ only when *C*_o_’s sample size exceeded a predetermined cutoff (*n*), determined by analyzing the pairwise differences between *C*_p_ and *C*_o_ relative to *N* (size of monthly ZCTA-level measurements). We also fitted simple linear regression models to explore the coefficient of determination (*R* ^2^) between *C*_p_ and *C*_o_, assessing the extent to which *C*_p_ could explain the variance of *C*_o_.

### Prediction

We used the final percentile-specific random forest models to generate monthly percentile predictions of ZCTA-level radon concentrations based on a full set of predictors. To isolate the effects of physical, environmental, and policy-related factors from artifacts of measurement practices, several adjustments were made during the prediction. First, the proportions of the four detector types were fixed at equal values (0.25) to remove spatial variation driven by regional differences in detector usage or performance. Also, policy-related predictors were standardized to prespecified reference levels, including mandatory radon disclosure requirements, no radon test programs, any state or federal measurement requirements, and high housing affordability. Second, we generated two sets of predictions by setting the proportion of basement measurements to 100% and 0%, yielding location-specific estimates for basements and above-ground floors. Third, instead of using meteorological conditions averaged over the exact radon measurement dates, we used population-weighted monthly averages to represent the typical monthly conditions. Finally, we obtained the predicted screening-level radon concentration for each monthly ZCTA by taking a weighted average of the basement and above-ground predictions using the predicted proportion of basement tests as the weight.

### Modeling Distribution

For each ZCTA and month, we fitted a log-normal distribution at the screening-floor level using the five predicted monthly percentiles, with parameters estimated via quantile matching using the “rriskDistributions” R package (version 2.1.2). We retained only the monotonic percentile sets (99.96% of predictions). Figure S3 illustrates the distribution of nonmonotonic predictions, with Ajo, Arizona exhibiting the highest number (159) of nonmonotonic percentiles. This emphasizes that the model demonstrates excellent performance across most regions, maintaining a strong monotonic relationship in the predictions across five percentiles. The log-mean and log-standard deviation were then derived as distributional parameters. The resulting monthly ZCTA-level probability density functions (PDFs) were defined over the range 0–3,700 Bq/m^3^. To characterize national and regional radon distributions, we aggregated ZCTA-level PDFs using population weights, such that each ZCTA contributed to the mixture distribution in proportion to its population size. The population-weighted PDFs were then integrated to generate the corresponding cumulative distribution functions. Based on these fitted parameters and distribution functions, we estimated the proportions of radon concentrations exceeding different thresholds (74, 148, and 370 Bq/m^3^) for each ZCTA, and summarized these exceedance probabilities across census regions, seasons, and study periods. To evaluate the adequacy of the log-normal approximation, we performed a goodness-of-fit check by comparing log-scale percentiles derived from the fitted log-normal distributions with log-scale percentiles calculated from the original radon measurements among ZCTA months.

### Uncertainty and Sensitivity Analyses

To quantify uncertainty in threshold exceedance probabilities, we conducted a bootstrap-based uncertainty analysis using the variance–covariance structure of cross-validated percentile residuals. We also conducted a lower-tail analysis to evaluate the influence of prediction error in the 10th percentile. Three sensitivity analyses were conducted to evaluate the robustness of the modeling framework. These included fitting percentile models without the resampling strategy, directly predicting ZCTA-month-specific log-normal parameters, and incorporating bootstrap-derived percentile uncertainty into the random forest training weights. Detailed methods are provided in Text S4.

## Results

### Spatial and Distributional Patterns of Predicted Radon Exposure

The proportions of ZCTAs with fitted screening-floor radon concentrations exceeding the EPA action level are summarized in Figure 1, and illustrative examples comparing fitted and measured ZCTA-level distributions are also shown in Figure S4. A total of 13,897 ZCTAs (48.9% of U.S. ZCTAs) had exceedance proportions above 20%, corresponding to at least one in five households being exposed to radon concentrations exceeding 148 Bq/m^3^. Among these ZCTAs, 7740 (55.7%) were in the Midwest, followed by 2954 (21.3%) in the Northeast. The South and West showed comparable counts, with 1747 (12.6%) and 1456 (10.5%) affected ZCTAs, respectively. Extending the analysis to the lower EPA-recommended action range (74–148 Bq/m^3^) revealed broader population coverage. A total of 25,002 ZCTAs (88.0%) were estimated to have >20% of households with radon concentrations exceeding 74 Bq/m^3^ (Figure S5).

**Figure 1:**
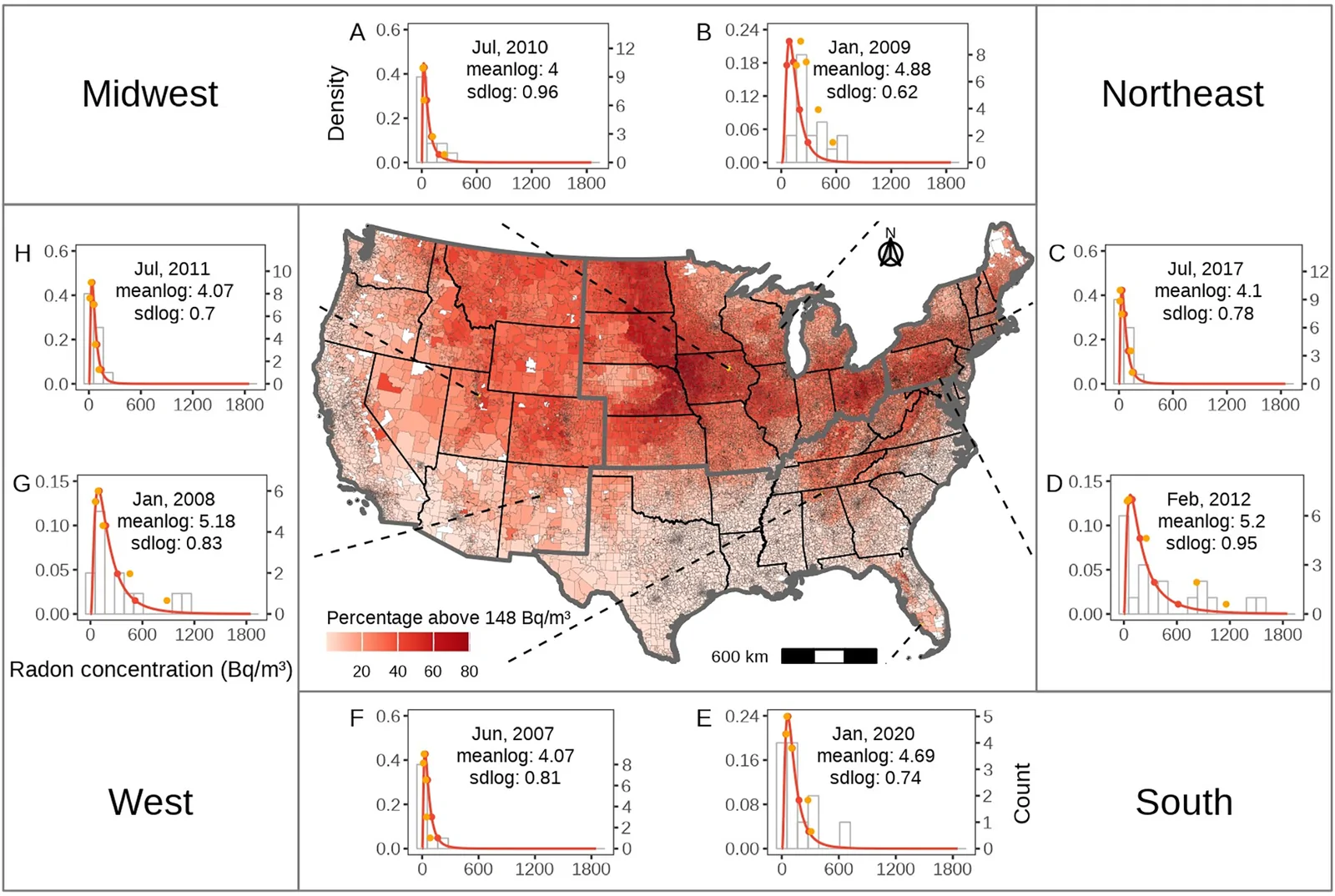
Percentage of predicted ZCTA-level indoor radon concentrations exceeding 148 Bq/m^3^ at the screening floor across the United States. Note: Panels (A–H) (labeled clockwise starting from the upper-left panel) show the distributions of indoor radon concentrations for eight illustrative locations (selected across regions and concentration settings; not restricted to the same year): (A), Cedar Falls, Iowa; (B), Green Bay, Wisconsin; (C), Sharon, Massachusetts; (D), York, Pennsylvania; (E), Bonita Springs, Florida; (F), Madison, Alabama; (G), Albuquerque, New Mexico; and (H), Salt Lake City, Utah. The red curve represents the log-normal distribution fitted to the predicted radon percentiles for each selected ZCTA. Red points indicate the corresponding percentiles derived from the fitted distribution, while orange points denote the 10th, 25th, 50th, 75th, and 90th percentiles derived from the measured radon concentrations. Histograms represent the distribution of radon measurements.

National and regional cumulative distributions of population-weighted radon concentrations are presented in Figure 2. The cumulative curves indicate a rapid increase in the proportion of the population exposed to radon levels above 74 Bq/m^3^, followed by a more gradual rise beyond the EPA action level of 148 Bq/m^3^, with the steepest increases observed in the Midwest. Nationally, 14.3% of the U.S. population (42.8 million people) is estimated to be exposed to radon concentrations exceeding 148 Bq/m^3^ (Table S2). Exposure above 74 Bq/m^3^ is substantially more widespread. 40.5% of the U.S. population (121.4 million people) resides in ZCTAs where radon concentrations exceed this threshold. This represents nearly three times the number of individuals exposed to above 148 Bq/m^3^. Notably, although the South has the lowest exceedance proportion above 74 Bq/m^3^ among regions, it accounts for a disproportionately large number of exposed individuals due to its larger population base. An estimated 34.5 million residents in the South exceed this threshold, second only to the Midwest (40.5 million) and exceeding the Northeast (23.9 million) and West (22.5 million) in absolute numbers.

**Figure 2:**
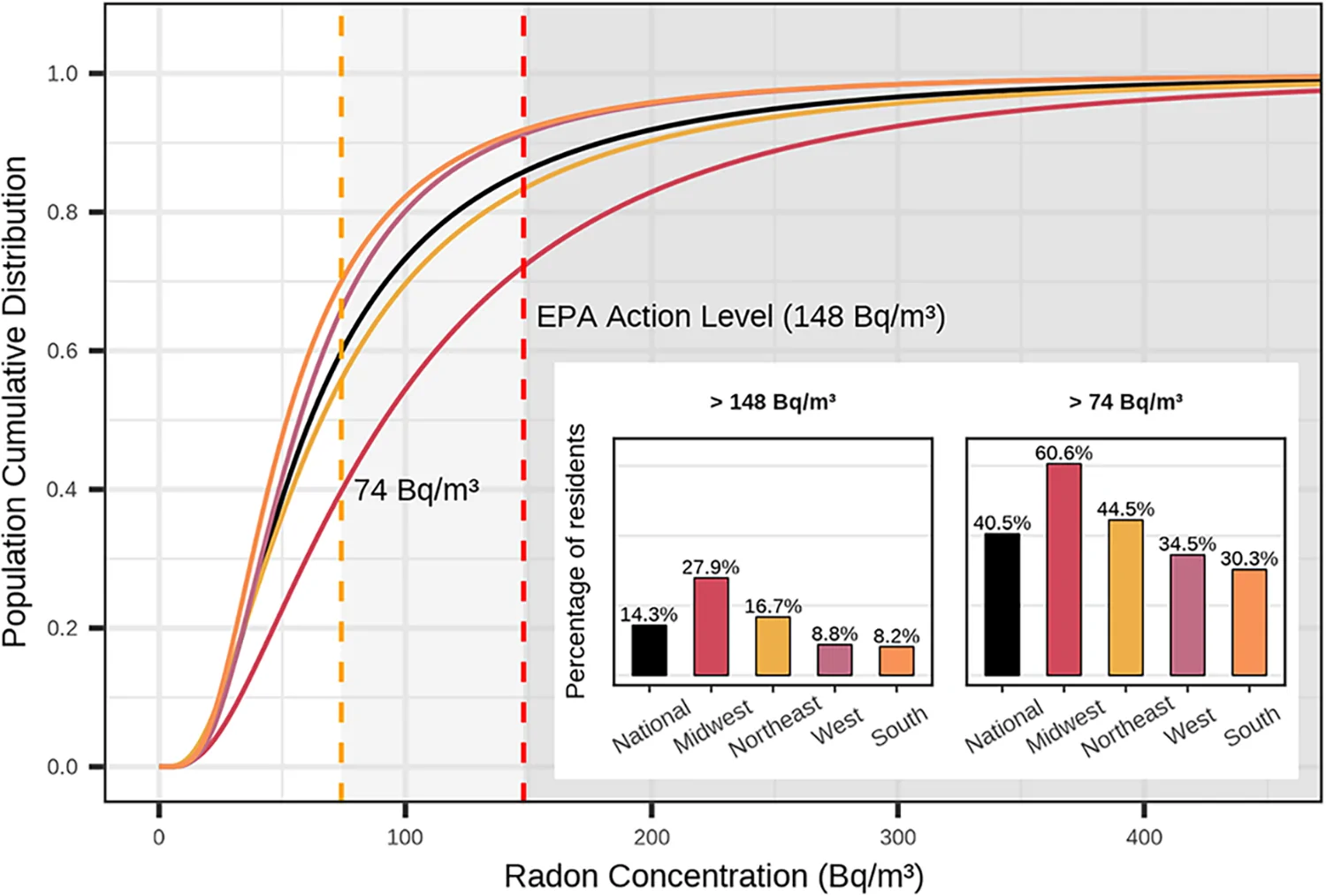
Cumulative distribution of population-weighted radon concentrations at the screening floor across U.S. regions.

The predicted population-weighted radon concentrations at the 10th, 25th, 50th, 75th, and 90th percentiles were 30.6, 43.4, 71.3, 114.1, and 169.2 Bq/m^3^, respectively (Figures 3 and S6). Across percentiles, the Midwest consistently exhibited the highest radon exposure. At the 90th percentile, population-weighted radon concentrations reached 245.7 Bq/m^3^ in the Midwest, followed by the Northeast (195.2 Bq/m^3^). Within-region dispersion varied across regions. The Northeast showed the highest average relative IQR (1.2), followed by the Midwest (1.1), reflecting greater heterogeneity and a higher likelihood of extreme concentrations (Figure S7). The fitted log-normal parameters show regional patterns consistent with those observed for the predicted percentiles and relative IQRs (Figures S8–S9).

**Figure 3:**
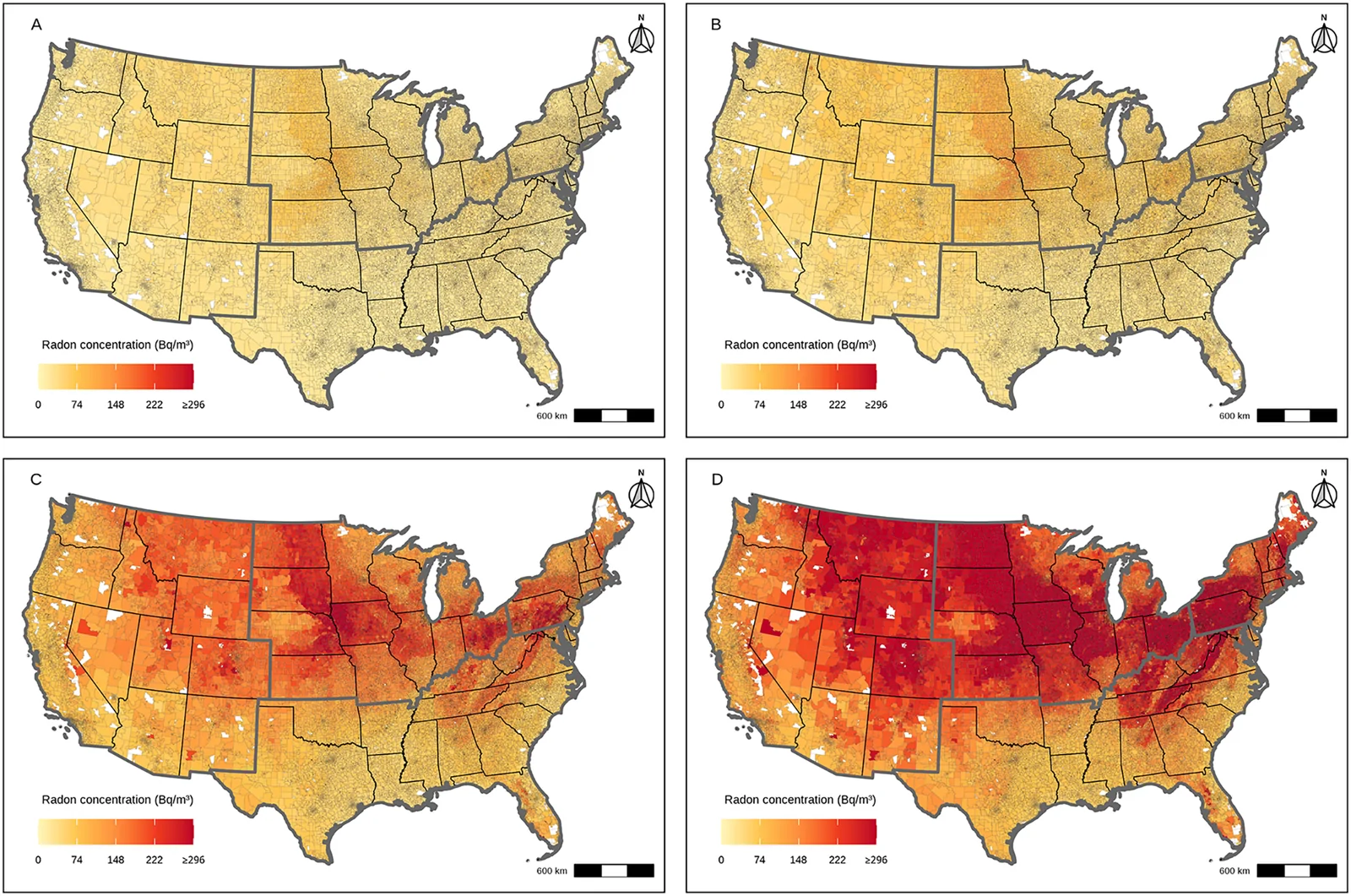
Spatial distribution of predicted ZCTA-level radon percentiles at the screening floor during 2000 to 2021. Panels (A–D) correspond to the 10th, 25th, 75th, and 90th percentiles of radon concentration, respectively.

### Temporal Trends in the Population Exposed to Radon Levels above the EPA Action Level

We further examined temporal changes in the population-weighted percentage of individuals exposed to radon concentrations above the U.S. EPA action level between 2000 and 2021, stratified by region and season (Figure 4). Across regions, exceedance percentages exhibited consistent seasonal patterns, with the highest proportions observed in winter and the lowest proportions in summer. Long-term trends in exceedance proportions varied by region, with larger declines observed in regions with higher radon exposure levels. The Midwest experienced the most pronounced decline, with an estimated decrease of approximately 0.06 percentage points per year, followed by the Northeast (0.05 percentage points per year) and West (0.01 percentage points per year). In contrast, no significant long-term decline was observed in the South over the study period.

**Figure 4:**
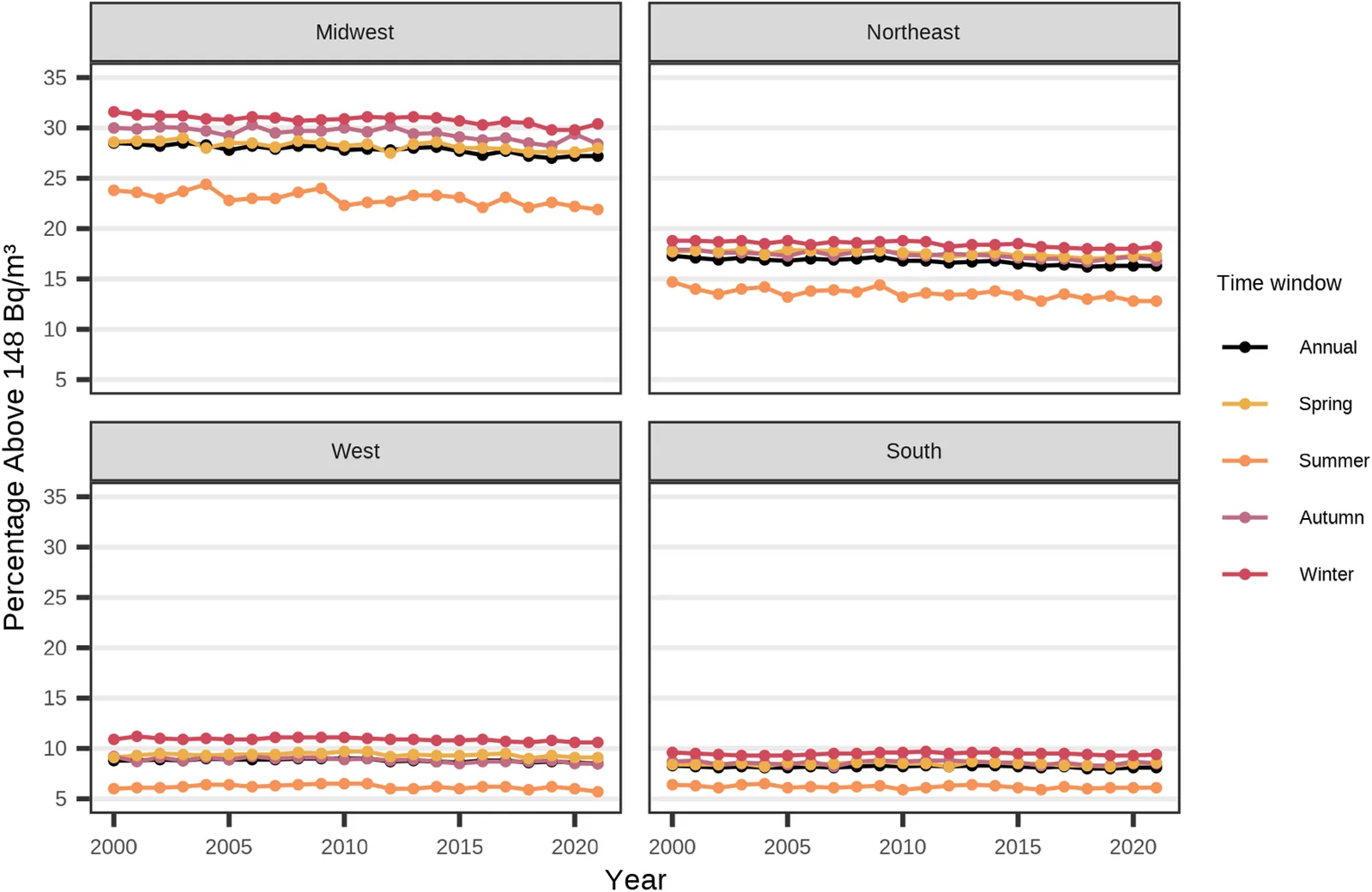
Annual and seasonal patterns of population-weighted radon exceedance above 148 Bq/m^3^ across U.S. regions, 2000–2021.

### Model Performance

The uncertainty of ZCTA-level radon measurements is a function of sample size: larger sample sizes generally yield smaller uncertainty. As shown in Figure S10, the 10-fold CV MAE decreases sharply as the sample size increases from 5 to 10, followed by smaller and less consistent improvements thereafter. Therefore, only MAEs based on large sample size (≥10) were used as reliable indicators of model accuracy. Prediction accuracy and bias also varied by the percentile of the radon distribution. For the lowest percentile (10th), the MRAE remained high at over 20% (Figure 5). Substantial improvement was observed at higher percentiles: when the sample size reached 25, MRAE values for the 25th-90th percentiles converged to below 20%, indicating minimal bias.

**Figure 5:**
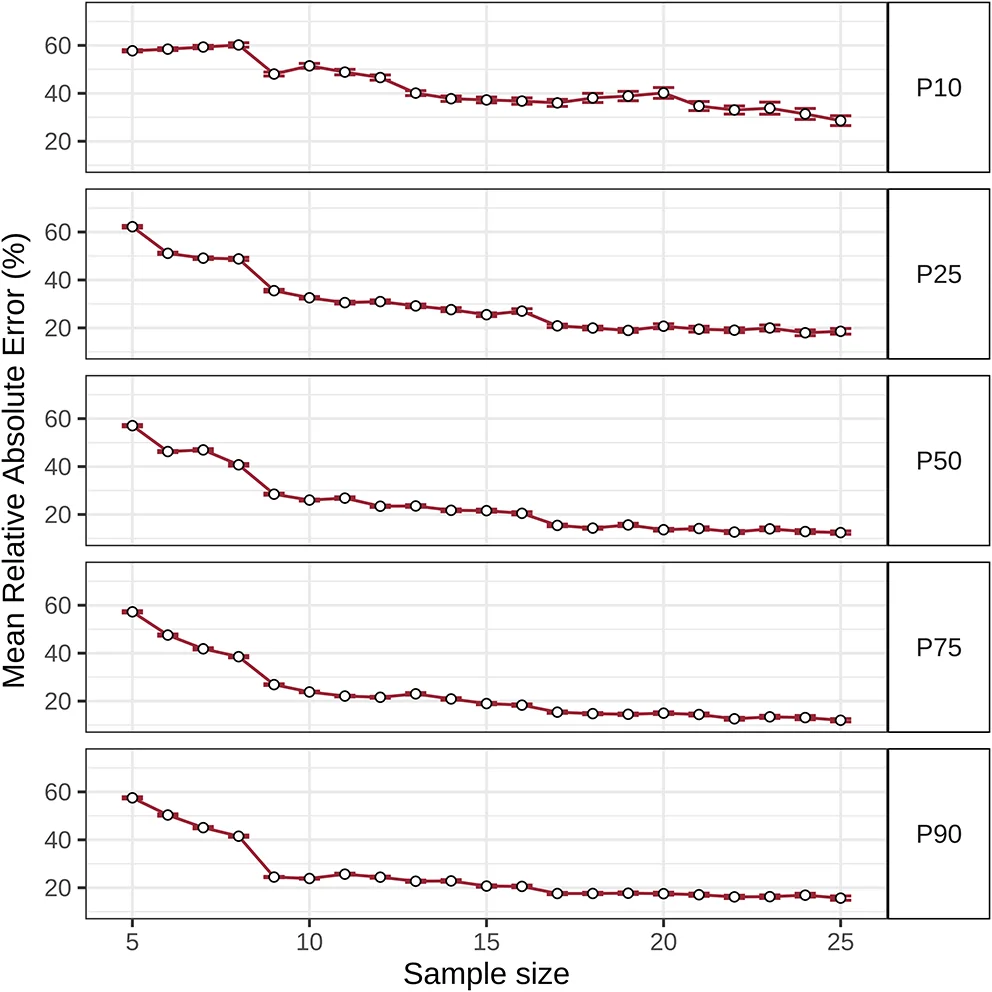
Trend of mean relative absolute error against sample size for the 10th, 25th, 50th, 75th, and 90th percentiles of radon concentrations based on 10-fold CV. Note: Error bars represent 95% confidence intervals for the mean relative absolute error, calculated using the standard error within each sample-size stratum.

Cross-validated *R* ^2^ on the log scale similarly demonstrated improved predictive performance with increasing sample size and higher radon percentiles (Figure S11). For the 25th, 50th, 75th, and 90th percentiles, *R* ^2^ ranged from 0.74 to 0.77 at sample sizes of 10–15 and consistently exceeded 0.85 when the sample size was ≥ 15, indicating strong predictive performance.

Goodness-of-fit check showed that *R* ^2^ values ranged from 0.57 to 0.61 among ZCTAs with 10–15 measurements and increased to 0.68–0.72 among ZCTAs with ≥15 measurements for the 50th, 75th, and 90th percentiles (Figure S12). These results suggest that the log-normal approximation captured the central and upper portions of the observed radon distribution reasonably well in ZCTAs with larger sample sizes. At higher observed concentrations, fitted values tended to be lower than observed values, suggesting that the fitted log-normal distribution may underrepresent the upper tail in some high-radon ZCTAs.

### Important Radon Predictors

At the category level (Figure 6), meteorological factors consistently produced the largest increases in prediction error upon permutation (100%), underscoring their dominant role across radon percentiles and regions. Different percentiles show distinct patterns of the important predictors. For the 10th percentile, meteorological and architectural factors were the two most significant predictors, contributing over 75%, while for the 90th percentile, meteorological, architectural, bedrock-related, and surficial factors emerged as the top predictors, each contributing over 40%. The relative importance of key factors across percentiles followed similar trends across the regions. Architectural and soil factors had the greatest influence at lower radon levels, with their importance decreasing at higher levels. In contrast, bedrock-related factors became more important at the upper tail of the radon distribution.

**Figure 6:**
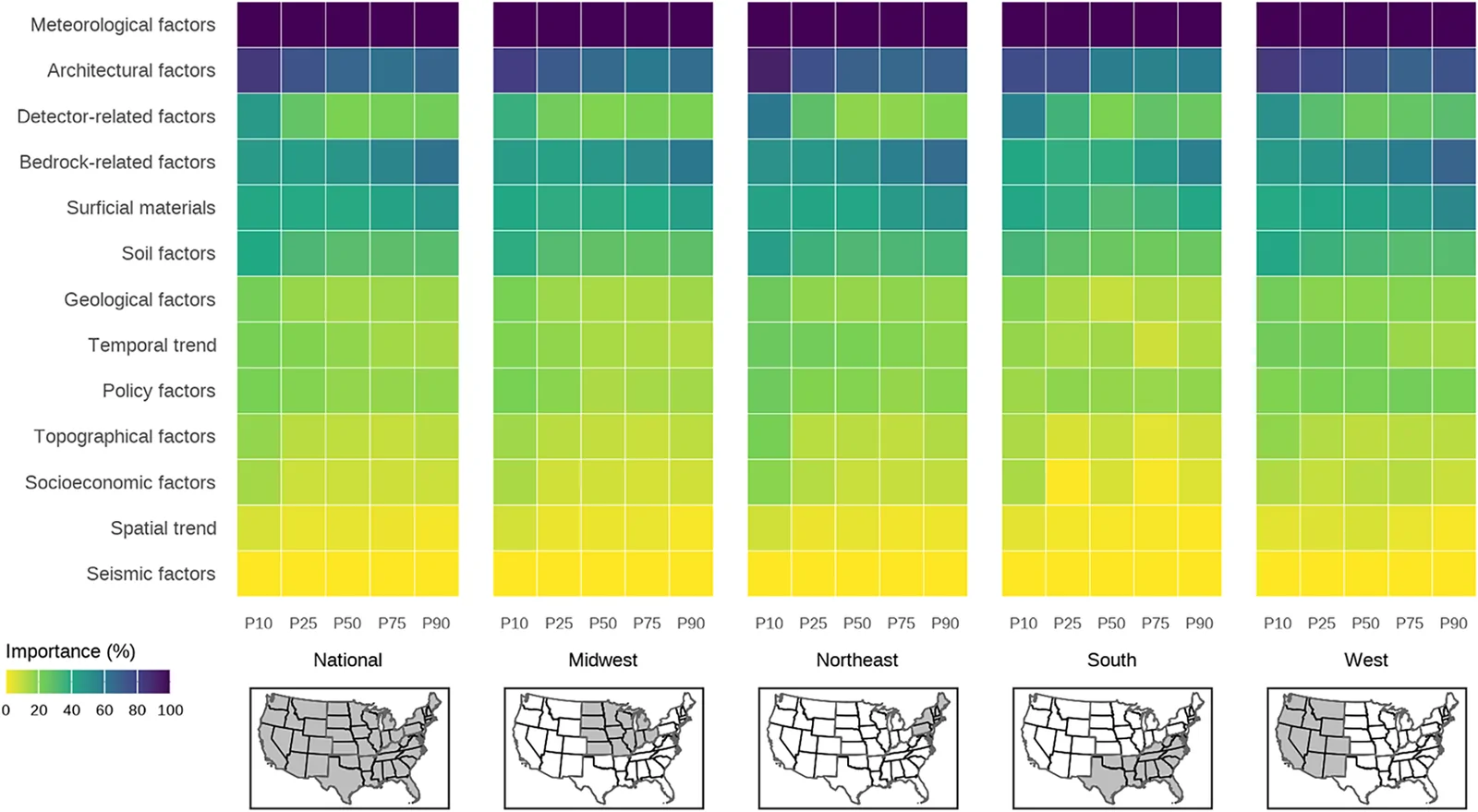
Variable importance by predictor category across radon percentiles.

### Uncertainty and Sensitivity Analyses

ZCTA-level exceedance probabilities showed substantial uncertainty, although uncertainty was markedly reduced after aggregation to regional and national levels (Figure S13 and Table S3). Increasing prediction error in the 10th percentile widened the uncertainty intervals but had little effect on median exceedance probabilities (Figure S14). Sensitivity analyses supported the use of the resampling-based percentile models, which generally showed better predictive performance than the alternative approaches (Table S4). Exceedance estimates varied under the parameter-based and uncertainty-weighted models, reflecting differences in how distributional parameters were modeled and percentile uncertainty was incorporated (Table S2). Detailed descriptions are provided in Text S5.

## Discussion

Our study expands understanding of the fine-grained distribution of indoor radon exposure across the U.S. by predicting ZCTA-level radon distributions over a 22-year period. By moving beyond area-level summary measures, our results provide a clear picture of the population radon exposure distribution and offer a robust foundation for targeted public health interventions and epidemiological research.

This study estimates that 14.3% of the U.S. population (42.8 million residents) is exposed to radon concentrations exceeding the U.S. EPA action level, approximately half of the estimate reported in our previous work,^11^ which was based on average predicted radon concentrations. By explicitly quantifying within-ZCTA distributional characteristics and constructing probability density functions of radon exposure, our approach substantially improves the estimation of the number and proportion of residents exceeding specific high-risk thresholds. For example, we estimated that 2.1% of the U.S. population (6.2 million people) is exposed to radon concentrations above 370 Bq/m^3^ (10 pCi/L), a level that is difficult to quantify reliably using mean- or median-based exposure estimates alone. Importantly, a population-based perspective reveals patterns that are obscured when focusing on exposure levels alone: although the South has the lowest exceedance proportion above the U.S. EPA action level among regions, it accounts for the second-largest number of exposed individuals because of its large population size. This distribution-based modeling framework therefore provides a flexible and quantitative approach for population-level risk assessment, highlighting the need to consider both the exposure intensity and the size of the affected population.

This study provided a detailed prediction of radon exposure across multiple percentiles, particularly at the 90th percentile. While the U.S. EPA recommends an action level of 148 Bq/m^3^, the spatial distribution of predicted radon concentrations at the 90th percentile reveals that extreme exposure levels exceeding 300 Bq/m^3^ (the threshold recommended by the WHO) are more common than those at the 50th percentile.^25^ This suggests that radon-related health risks may be substantially underestimated for individuals in higher exposure categories, as area-level classifications based on a single action level can obscure meaningful differences between those experiencing extreme exposures and concentrations just above the U.S. EPA action level. Our study further enhances our understanding of exposure variability within U.S. ZCTAs. Given the highly right-skewed nature of the radon distribution, the spatial patterns of radon concentrations at higher percentiles are strongly influenced by both the median level and variability in exposure. The predictions at these high percentiles underscore the importance of considering this variability, highlighting areas with extreme radon exposure such as Naples, Florida, and Beacon Falls, Connecticut. These areas, while not classified as high-radon ZCTAs based on median concentrations, exhibit high relative IQRs (greater than 2), indicating significant exposure risks that are not reflected by median levels alone (Figure S15).

It is estimated that 6% of U.S. housing units are exposed to radon concentrations above the EPA action level from the national residential radon survey.^26^ However, such summary statistics may not sufficiently motivate the public to conduct radon testing, as this is a sampling survey. These findings suggest the need to shift the focus of population-level risks from national or regional levels to the small area level, allowing for more targeted and precise interventions.^27^ Our findings indicate that in nearly half of all ZCTAs, at least one in five households is exposed to radon levels exceeding the U.S. EPA action level. Our model provides the public with detailed estimates of the proportion of households in their own ZCTAs that are exposed to radon concentrations above the action level. This localized information provides a stronger incentive for individuals to take proactive measures such as testing and mitigation. Moreover, our findings support the need for government agencies to focus on high-risk ZCTAs rather than on broader state-level regions. By implementing more proactive and cost-effective radon testing initiatives, resources can be allocated efficiently to areas where they are most needed.

We observed that across regions, several predictor domains were consistently important for the upper percentile, including meteorological, architectural, bedrock-related, surficial-material, and soil-related factors. These domains capture key components of the radon exposure pathway and are consistent with prior literature identifying geology, soil permeability, building foundation characteristics, and meteorological conditions as major drivers of high indoor radon.^28–31^ Many dominant predictors, such as bedrock, surficial geology, and soil properties, are upstream environmental determinants that are not directly modifiable at the household level. However, the relative importance of these domains varied across regions, indicating that local environmental and housing characteristics still contribute to small-area differences in radon distributions. At the same time, we observed decreasing temporal trends in exceedance probabilities above the EPA action level in the Midwest and Northeast. These patterns may be consistent with long-term radon risk reduction efforts, including increased testing, mitigation of existing homes, and radon-resistant construction.^32^ From a spatiotemporal perspective, our findings suggest that upper-tail indoor radon concentrations reflect both persistent environmental determinants and potentially modifiable processes, but the results should primarily be used to support risk identification, targeted testing, and public health communication rather than to infer specific intervention effects.

Our uncertainty analysis highlights the need to interpret ZCTA-level exceedance probabilities with caution. Because exceedance probabilities are derived from fitted log-normal distributions, they are sensitive to the fit of the upper tail, particularly when the separately predicted percentiles do not perfectly conform to a single log-normal distribution. The bootstrap analysis showed that ZCTA-level exceedance probabilities had substantial uncertainty, especially for tail-dependent estimates. However, model performance and goodness of fit improved with increasing sample size, indicating greater reliability in ZCTAs with more observations. Therefore, local exceedance estimates should be interpreted together with the sample size and uncertainty information. Importantly, uncertainty was markedly reduced after population-weighted aggregation, and regional and national exceedance estimates were robust across the bootstrap replicates. Despite local uncertainty, small-area estimates remain valuable for epidemiological exposure assessment and for identifying areas where targeted radon testing and mitigation may be warranted.

Another important consideration in interpreting the findings is the nonprobability nature of the radon measurements. Previous studies have shown that nonrandom testing can introduce selection bias and may lead to overestimation of indoor radon concentrations.^33–36^ Although the measurements in our study were collected during property transactions, testing may still be influenced by disclosure requirements, radon awareness, prior concern about elevated radon, and financial resources.^37^ Tested homes may also differ from the broader housing stock in heating, ventilation, and air conditioning (HVAC) systems and other housing characteristics. These mechanisms may affect both within-ZCTA estimates and the geographic representativeness of the national samples. To partially address these sources of bias, we retained only the first measurement for addresses with multiple tests because subsequent measurements may have been conducted to confirm the need for mitigation or to evaluate the mitigation effectiveness. We also incorporated policy-related predictors to account for geographic variation in testing requirements and housing market conditions and standardized these variables during prediction to reduce the influence of measurement selection and implementation processes.

Several limitations exist in our study. First, selection bias may remain because state- and county-level predictors could be too coarse to capture local testing behavior, and detailed information about mitigation status, test operators, and HVAC systems was unavailable. Residual selection bias may differ across states, regions, and population density contexts, potentially overrepresenting higher-risk homes or areas and leading to overestimation of upper percentiles and threshold exceedance probabilities in some ZCTAs. Second, the prediction accuracy was lower for the 10th percentile of radon concentrations. One possible reason is that many low-level radon measurements fall near or below the detection limits of the detectors. Imputation of these left-censored values introduces additional uncertainty mainly for lower-tail percentile estimates, especially the 10th and 25th percentiles, while having a less direct influence on the median and upper percentiles. Additionally, areas with lower radon concentrations tend to have fewer measurement records, as radon testing is less frequently mandated in these regions.^38^ Together, these factors make low-end exposure levels inherently more difficult to predict. However, the strong monotonicity across the five percentiles and the relatively accurate predicted distributions suggest that, despite challenges in predicting low-level concentrations, the model offers valuable insights into identifying spatial variation. Third, the lack of data on individual building characteristics prevents the prediction of radon concentrations at the building level, presenting a challenge in accurately capturing the full distribution of radon concentrations. Radon concentrations are highly sensitive to localized building features, such as HVAC systems and building airtightness, as well as local geological factors, particularly for small areas. Nevertheless, the study incorporated an extensive set of predictors (189 variables) and made predictions at a fine spatial resolution (ZCTAs), providing strong evidence to support the population-level risk identification and communication. Fourth, the study did not account for above-ground-floor-dependent gradients in multifamily buildings, which are increasingly common in densely populated urban areas. However, the available data include only floor-level information for single-family homes. Previous research has shown that the variation in radon concentrations between above-ground floors is generally smaller than the difference between basements and above-ground floors.^39^ Therefore, aggregating above-ground measurements regardless of building type is not expected to be a dominant source of bias in ZCTA-level observations, although uncertainty introduced by missing floor-level data for multifamily buildings warrants further quantification. Fifth, each ZCTA-month radon distribution was approximated using a single log-normal distribution. Although this assumption is consistent with the positive and right-skewed nature of indoor radon concentrations and was supported by a goodness-of-fit check, it may not fully capture complex within-ZCTA heterogeneity. Some ZCTAs may contain mixed housing types, such as single-family homes with basements and multiunit apartment buildings, which could lead to departures from a single log-normal distribution or potentially multimodal patterns. More flexible distributional approaches would require estimating additional parameters and would therefore require larger within-ZCTA sample sizes, which were not consistently available in our study. Sixth, we used 2015 ZIP codes as the harmonized spatial reference and linked ZIP-based radon measurements to ZCTA characteristics using identical five-digit codes. Because ZIP codes and ZCTAs are not identical and may change over time, this linkage may introduce spatial misclassification that cannot be fully quantified. Finally, the percentile-specific models were fitted separately and therefore did not account for the natural dependence among percentiles. Future work should consider joint or constrained modeling strategies that preserve both dependence and monotonicity across the predicted percentiles.

This national study offers significant advancements in radon distribution mapping by predicting multiple percentiles, providing a clearer and more detailed understanding of distribution characteristics in radon levels within ZCTAs in the U.S. Our analysis effectively captures extreme high-exposure areas, which are often overlooked when considering only average concentrations. Additionally, our method is capable of calculating the excess distribution of radon exposure on a monthly basis at the ZCTA level. This finer temporal and spatial resolution offers a dynamic view of how radon levels fluctuate over time, potentially influencing health risks associated with short-term or prenatal exposure. Another key innovation is the creation of a comprehensive database. This enriched data set serves as a valuable resource for policymakers, enabling the design of targeted interventions such as ZCTA-specific regulations for passive mitigation in new buildings. It also provides health researchers with an essential tool for examining radon-related health effects by incorporating within unit exposure heterogeneity into health analyses.

## Supplementary Material



## Data Availability

The ZCTA-level indoor radon exceedance probabilities and corresponding uncertainty information generated in this study are publicly available at an Open Science Framework (OSF) repository (DOI: 10.17605/OSF.IO/3SHC4). All model codes are available at a GitHub repository https://github.com/longxiang1025/Radon_Mortality (55). Summary data and additional results are provided in the main text and Supporting Information.

## Notes

Left-censored modeling approach, radon predictors, categorical variable importance, and uncertainty and sensitivity analyses; sources and resolutions of radon predictors; population-weighted threshold exceedances and regional probabilities; spatial distributions of measured and predicted radon concentrations; model performance and cross-validation results; and prediction uncertainty and exposure heterogeneity (PDF)
