# Supplementary material for: From Percentiles to Distributions: Nationwide ZCTA-Level Modeling of Indoor Radon Exposure across the Contiguous United States, 2000–2021
Source: Environ Sci Technol. 2026 Aug 31;60(36):25597–606. doi: 10.1021/acs.est.6c01333 (PMC13585067; doi:10.1021/acs.est.6c01333)
Supplement: Supplementary file 1 [file es6c01333_si_001.pdf]

Supplementary Materials

From Percentiles to Distributions: Nationwide ZCTA-Level Modeling of Indoor Radon Exposure Across the Contiguous United States, 2000–2021

Teng Yang [a], Longxiang Li [a*]

[a] Gangarosa Department of Environmental Health, Rollins School of Public Health, Emory University, Atlanta, Georgia 30322, United States

Corresponding Author: Longxiang Li −Gangarosa Department of Environmental Health, Rollins School of Public Health, Emory University, Atlanta, Georgia, 30322 United States; orcid.org/0000-0003-0893-146X; Email: lli224@emory.edu

This file includes:

Number of pages: 35

Number of supporting texts: 5

Number of tables: 4

Number of figures: 15

Table of Contents

Text S1. Maximum likelihood–based left-censored approach 4

Text S2. Description of Radon Predictors 4

Text S3. Categorical Variable Importance Approach 4

Text S4. Uncertainty and Sensitivity Analyses Methods 5

Text S5. Uncertainty and Sensitivity Analyses Results 6

Table S1. Sources and resolutions of radon predictors 7

Table S2. Population-weighted percentages and numbers of residents exceeding selected indoor radon thresholds derived from different training models 14

Table S3. Population-weighted threshold exceedance probabilities and bootstrap standard-error-based 95% CI by region. 15

Table S4. MAE (Log-transformed) for the 10th, 25th, 50th, 75th, and 90th percentiles of radon concentrations based on 10-fold CV derived from different training models. 16

Fig. S1. Density distribution of national log-transformed indoor radon measurements. 17

Fig. S2. Regional distributions of log-transformed indoor radon measurements. 18

Fig. S3. Spatial distribution of ZCTAs with non-monotonic predicted radon percentiles. 19

Fig. S4. Comparisons between fitted and measured ZCTA-level indoor radon concentrations on log scale. 20

Fig. S5. Percentage of predicted ZCTA-level indoor radon concentrations exceeding 74 Bq/m³ at the screening floor across the United States. 21

Fig. S6. The median of average predicted ZCTA-level radon concentrations at the screening floor from 2000 to 2021. 22

Fig. S7. The relative interquartile range (IQR) of average predicted ZCTA-level radon concentrations at the screening floor from 2000 to 2021. 23

Fig. S8. Spatial distribution of the long-term mean of log-transformed predicted radon concentration (meanlog) at the screening floor averaged over 2000–2021. 24

Fig. S9. Spatial distribution of the long-term standard deviation of log-transformed predicted radon concentration (sdlog) at the screening floor averaged over 2000–2021 25

Fig. S10. Trend of MAE (Log-transformed) against sample size for the 10th, 25th, 50th, 75th, and 90th percentiles of radon concentrations based on 10-fold CV. 26

Fig. S11. The scatter plots between the predicted and observed radon concentrations by percentiles, stratified by the sample size of observation, according to the results of 10-fold cross validation. 27

Fig. S12. The scatter plots between the fitted and observed radon concentrations by percentiles, stratified by the sample size of observation. 29

Fig. S13. Comparison of bootstrap-based and fitted exceedance probabilities in Pennsylvania in 2015 across different thresholds. 31

Fig. S14. Lower-tail uncertainty analysis for exceedance probabilities above 148 Bq/m³ in Pennsylvania in 2015 across different errors. 32

Fig. S15. Measured and fitted distributions of indoor radon concentrations illustrating within-ZCTA exposure heterogeneity during 2000-2021 in selected ZCTAs. 33

Text S1. Maximum likelihood–based left-censored approach

Radon measurements were obtained using four types of charcoal based passive detectors: the Air Chek foil bag charcoal kit (AirChek), the AccuStar charcoal canister (AccuStar PicoCan), liquid scintillation vials (AccuStar CLS), and the Alpha Energy tray (Alpha Energy). Measurements below the detector-specific LDLs were treated as left-censored observations. The detector-specific LDLs were 14.8 Bq/m³ for AirChek and Alpha Energy detectors and 18.5 Bq/m³ for AccuStar PicoCan and CLS detectors. The LDL is a counting statistic, defined as the smallest net count rate at which there is 95% confidence that a signal above background is detected.[1] To impute values below the LDLs using a maximum likelihood–based left-censored approach, a lognormal distribution was fitted to both detected and left-censored measurements, and below-LDL values were imputed by random sampling from the fitted distribution truncated below the corresponding LDL.

Text S2. Description of Radon Predictors

Detector-related variables accounted for measurement heterogeneity due to detector types and geographic differences in detector use. Systematic differences across detector types may arise from laboratory-specific protocols used to address real-world measurement conditions that are not fully controlled in chamber testing, such as temporal fluctuations in radon concentrations, airflow, and barometric pressure.[2,3] Geological predictors represented radon generation and soil-gas transport processes,[4] such as radon potential, and bedrock and surficial geology.[5,6] Architectural predictors characterized pathways for soil-gas infiltration [7] and other indoor sources, such as building characteristics and energy-use patterns.[8,9] Socioeconomic indicators reflected factors that influence energy efficiency and the prevalence of radon mitigation.[10] Meteorological predictors captured short-term conditions affecting soil-gas exhalation, subsurface radon movement, and atmospheric radon concentrations. [6,11-15] To capture potential measurement selection and implementation processes, policy-related predictors state-level radon disclosure requirements, radon test programs, measurement personnel requirements, and county-level housing affordability were included.[16,17] To derive percentiles of meteorological predictors, daily meteorological variables were first interpolated onto 30 arc-second grids based on the Gridded Population of the World, Version 4 (GPWv4).[18] The variables were then averaged over the specific days corresponding to the radon measurement period within each month and subsequently aggregated to ZCTA boundaries using population-weighted averaging. This approach allowed the meteorological predictors to capture meteorological conditions during the measurement period.

For geological, architectural, and socioeconomic predictors, which represent single-time measurements, population-weighted percentiles were directly extracted and calculated without temporal averaging.

Text S3. Categorical Variable Importance Approach

For each category, all predictors within that category were randomly permuted simultaneously, while predictors in other categories were left unchanged. The random forest model was then refit

using the same cross-validation folds, and category-level importance was quantified by the increase in MAE compared with the original model without permutation. Importance scores were normalized using min–max scaling to facilitate comparability across categories and radon percentiles. This grouped approach provides a more stable summary of category-level contributions than individual predictor-level importance alone.

Text S4. Uncertainty and Sensitivity Analyses Methods

For uncertainty analyses, random errors were added to the predicted percentiles based on the variance–covariance matrix of cross-validated residuals between observed and predicted radon percentiles, estimated among ZCTA-months with at least 10 measurements. For each bootstrap replicate, the perturbed percentiles were used to refit the lognormal distribution, and threshold exceedance probabilities above 74, 148, and 370 Bq/m^3 were recalculated. This procedure was repeated for 100 bootstrap replicates. We summarized ZCTA-level uncertainty using the width of the 95% bootstrap confidence interval (CI) and further aggregated bootstrap estimates to regional and national levels using population weights. Bootstrap standard-error-based 95% CI were calculated for regional and national population-weighted exceedance probabilities using the standard deviation of the bootstrap estimates.

Because the MRAE for the 10th percentile remained relatively high, we conducted an additional lower-tail uncertainty analysis using Pennsylvania in 2015 to evaluate the influence of lower-tail error on exceedance probabilities. Specifically, we perturbed only the predicted 10th percentile using cross-validated residual errors under three scenarios: a small-error scenario based on residuals from ZCTA-months with sample size ≥20, a medium-error scenario based on sample size ≥10, and a large-error scenario based on sample size ≥5. For each replicate, the lognormal distribution was then refitted and the threshold exceedance probability above 148 Bq/m^3 was recalculated and summarized across the three scenarios.

Three sensitivity analyses were conducted. First, because percentile-based targets may be sensitive to sample size and to the specific observations included within each ZCTA-month, we conducted a sensitivity analysis by training a benchmark random forest model without the resampling strategy. Second, we used a parameter-based modeling approach to evaluate an alternative strategy that avoids potential non-monotonicity in separately predicted percentiles. We first estimated ZCTA-month-specific lognormal parameters, including the log-scale mean and standard deviation, from the observed radon measurements. We then trained separate random forest models to predict these two lognormal parameters using population-weighted means and standard deviations of the predictor variables as input features, instead of using population-weighted predictor percentiles. Third, to evaluate the influence of uncertainty in percentiles during model training, we conducted an uncertainty-weighted sensitivity analysis. For each ZCTA-month, we estimated the uncertainty of each percentile using a bootstrap procedure and calculated the corresponding standard deviation.

We then incorporated the inverse of this bootstrap-derived standard deviation as an additional component of the random forest training weight.

Text S5. Uncertainty and Sensitivity Analyses Results

To illustrate uncertainty in threshold exceedance probabilities arising from prediction errors in the fitted percentile-based distributions, we used Pennsylvania in 2015 as an example (**Fig. S13**). The median exceedance probability above 148 Bq/m³ was 0.326, with a median 95% bootstrap CI width of 0.350. For the 370 Bq/m³ threshold, the median exceedance probability was 0.067, with a median 95% bootstrap CI width of 0.167. These results indicate substantial uncertainty in ZCTA-level exceedance probabilities. However, uncertainty was substantially reduced after aggregation to larger spatial units. The bootstrap standard-error-based 95% CI for the population-weighted exceedance probability above 148 Bq/m³ was narrow at the national level (14.26%–14.31%) and across regions (**Table S3**).

The lower-tail uncertainty analysis showed that larger perturbations of the 10th percentile increased uncertainty but had little effect on the median exceedance probability above 148 Bq/m³ (**Fig. S14**). The median exceedance probability remained approximately 0.33 across the small-, medium-, and large-error scenarios, with median 95% bootstrap confidence interval widths of 0.018, 0.027, and 0.046, respectively. Across all three scenarios, bootstrap exceedance probabilities were generally centered around the original fitted exceedance probabilities, with no clear systematic shift, although dispersion increased under the larger-error scenario.

The benchmark model without resampling showed poorer predictive performance across all percentile targets than the main resampling-based model, with log-scale MAEs of 0.403, 0.337, 0.298, 0.289, and 0.326 for the 10th, 25th, 50th, 75th, and 90th percentiles, respectively (**Table S4**). However, the population-weighted exceedance probability above 148 Bq/m³ was comparable between the benchmark and main models at the national and regional level. In the parameter-based sensitivity analysis, the estimated population-weighted exceedance probability above 148 Bq/m³ was higher than that from the main percentile-based model nationally (17.0% vs. 14.3%) and across all four regions (**Table S2**). However, this approach introduced additional uncertainty in estimating distributional characteristics because direct prediction of the lognormal standard deviation parameter was challenging. The uncertainty-weighted sensitivity analysis improved prediction for the lower percentiles but worsened performance for the central and upper percentiles. The log-scale MAEs were 0.304, 0.232, 0.210, 0.228, and 0.307 for the 10th, 25th, 50th, 75th, and 90th percentiles, respectively (**Table S4**). The uncertainty-weighted model produced lower population-weighted exceedance probabilities above 148 Bq/m³ than the main model, with a national estimate of 10.0% compared with 14.3% in the main model (**Table S2**). Regional estimates were also lower. This pattern likely reflects downweighting of high-concentration ZCTA-months, where upper-tail percentile estimates had greater bootstrap uncertainty.

Table S1. Sources and resolutions of radon predictors.

No.	Covariate	Type	Resolution (Spatial/Temporal)	Source
1	Percent of measurements in the basement	Detector-dependent	ZCTA/Monthly	Laboratories
2	Percent of measurements by AccuStar Charcoal Canister	Detector-dependent	ZCTA/Monthly	Laboratories
3	Percent of measurements by AccuStar Liquid Scintillation	Detector-dependent	ZCTA/Monthly	Laboratories
4	Percent of measurements by AirChek Foil Bag	Detector-dependent	ZCTA/Monthly	Laboratories
5	Percent of measurements by Alpha Energy detector	Detector-dependent	ZCTA/Monthly	Laboratories
6	Ground surface concentration of U-238	Geological	3 km/Invariant	U.S. Geological Survey Radiometric Map
7	Geological radon potential	Geological	Geological province/Invariant	U.S. Geological Survey
8	Magnetic anomaly	Geological	0.05 degree/Invariant	U.S. Geological Survey
9	Gravity Anomaly (isostatic residual)	Geological	0.05 degree/Invariant	U.S. Geological Survey
10	Gravity Anomaly (Bouguer anomaly)	Geological	0.05 degree/Invariant	U.S. Geological Survey
11	Distance to geological fault	Seismic	Geological line/Invariant	U.S. Geological Survey Data Series 424
12	Density of geological fault	Seismic	0.05 degree/Invariant	U.S. Geological Survey Data Series 424
13	Density of growth fault	Seismic	0.05 degree/Invariant	U.S. Geological Survey Data Series 424
14	Density of thrust fault	Seismic	0.05 degree/Invariant	U.S. Geological Survey Data Series 424
15	Density of strike fault	Seismic	0.05 degree/Invariant	U.S. Geological Survey Data Series 424
16	Density of normal fault	Seismic	0.05 degree/Invariant	U.S. Geological Survey Data Series 424
17	Elevation	Topographic	1 km/Invariant	U.S. Geological Survey National Elevation Dataset
18	Terrain slope	Topographic	1 km/Invariant	U.S. Geological Survey National Elevation Dataset
19	Topographic Ruggedness Index (TRI)	Topographic	1 km/Invariant	U.S. Geological Survey National Elevation Dataset
20	Topographic Position Index (TPI)	Topographic	1 km/Invariant	U.S. Geological Survey National Elevation Dataset
21	Roughness	Topographic	1 km/Invariant	U.S. Geological Survey National Elevation Dataset
22	Percent over alluvial surficial materials	Surficial Materials	ZCTA/Invariant	U.S. Geological Survey Data Series 425
23	Percent over coastal surficial materials	Surficial Materials	ZCTA/Invariant	U.S. Geological Survey Data Series 425
24	Percent over calcareous surficial materials	Surficial Materials	ZCTA/Invariant	U.S. Geological Survey Data Series 425

25	Percent over organic surficial materials	Surficial Materials	ZCTA/Invariant	U.S. Geological Survey Data Series 425
26	Percent over glacial surficial materials	Surficial Materials	ZCTA/Invariant	U.S. Geological Survey Data Series 425
27	Percent over glaciofluvial surficial materials	Surficial Materials	ZCTA/Invariant	U.S. Geological Survey Data Series 425
28	Percent over proglacial surficial materials	Surficial Materials	ZCTA/Invariant	U.S. Geological Survey Data Series 425
29	Percent over lacustrine surficial materials	Surficial Materials	ZCTA/Invariant	U.S. Geological Survey Data Series 425
30	Percent over eolian surficial materials	Surficial Materials	ZCTA/Invariant	U.S. Geological Survey Data Series 425
31	Percent over Massmovement surficial materials	Surficial Materials	ZCTA/Invariant	U.S. Geological Survey Data Series 425
32	Percent over residual surficial materials	Surficial Materials	ZCTA/Invariant	U.S. Geological Survey Data Series 425
33	Percent over water surficial materials	Surficial Materials	ZCTA/Invariant	U.S. Geological Survey Data Series 425
34	Percent over rock surficial materials	Surficial Materials	ZCTA/Invariant	U.S. Geological Survey Data Series 425
35	Percent over surficial materials with min age of late Pleistocene	Surficial Materials	ZCTA/Invariant	U.S. Geological Survey Data Series 425
36	Percent over surficial materials with min age of Holocene	Surficial Materials	ZCTA/Invariant	U.S. Geological Survey Data Series 425
37	Percent over surficial materials with max age of early Pleistocene	Surficial Materials	ZCTA/Invariant	U.S. Geological Survey Data Series 425
38	Percent over surficial materials with max age of late Pleistocene	Surficial Materials	ZCTA/Invariant	U.S. Geological Survey Data Series 425
39	Percent over surficial materials with max age of middle Pleistocene	Surficial Materials	ZCTA/Invariant	U.S. Geological Survey Data Series 425
40	Percent over surficial materials with max age of Pleistocene	Surficial Materials	ZCTA/Invariant	U.S. Geological Survey Data Series 425
41	Percent over surficial materials with max age of Pliocene	Surficial Materials	ZCTA/Invariant	U.S. Geological Survey Data Series 425
42	Percent over thin surficial materials	Surficial Materials	ZCTA/Invariant	U.S. Geological Survey Data Series 425
43	Percent over thick surficial materials	Surficial Materials	ZCTA/Invariant	U.S. Geological Survey Data Series 425
44	Percent over patchy surficial materials	Surficial Materials	ZCTA/Invariant	U.S. Geological Survey Data Series 425
45	Percent over bedrock with max age of Precambrian	Bedrock	ZCTA/Invariant	U.S. Geological Survey the State Geologic Map Compilation
46	Percent over bedrock with max age of Devonian	Bedrock	ZCTA/Invariant	U.S. Geological Survey the State Geologic Map Compilation
47	Percent over bedrock with max age of Silurian	Bedrock	ZCTA/Invariant	U.S. Geological Survey the State Geologic Map Compilation
48	Percent over bedrock with max age of Tertiary	Bedrock	ZCTA/Invariant	U.S. Geological Survey the State Geologic Map Compilation

49	Percent over bedrock with max age of Quaternary	Bedrock	ZCTA/Invariant	U.S. Geological Survey the State Geologic Map Compilation
50	Percent over bedrock with max age of Paleozoic	Bedrock	ZCTA/Invariant	U.S. Geological Survey the State Geologic Map Compilation
51	Percent over bedrock with max age of Cretaceous	Bedrock	ZCTA/Invariant	U.S. Geological Survey the State Geologic Map Compilation
52	Percent over bedrock with max age of Carboniferous	Bedrock	ZCTA/Invariant	U.S. Geological Survey the State Geologic Map Compilation
53	Percent over bedrock with max age of Ordovician	Bedrock	ZCTA/Invariant	U.S. Geological Survey the State Geologic Map Compilation
54	Percent over bedrock with max age of Jurassic	Bedrock	ZCTA/Invariant	U.S. Geological Survey the State Geologic Map Compilation
55	Percent over bedrock with max age of Triassic	Bedrock	ZCTA/Invariant	U.S. Geological Survey the State Geologic Map Compilation
56	Percent over bedrock with max age of Permian	Bedrock	ZCTA/Invariant	U.S. Geological Survey the State Geologic Map Compilation
57	Percent over bedrock with max age of Neoproterozoic	Bedrock	ZCTA/Invariant	U.S. Geological Survey the State Geologic Map Compilation
58	Percent over bedrock with max age of Paleoproterozoic	Bedrock	ZCTA/Invariant	U.S. Geological Survey the State Geologic Map Compilation
59	Percent over bedrock with max age of Mesoproterozoic	Bedrock	ZCTA/Invariant	U.S. Geological Survey the State Geologic Map Compilation
60	Percent over bedrock with max age of Mesozoic	Bedrock	ZCTA/Invariant	U.S. Geological Survey the State Geologic Map Compilation
61	Percent over bedrock with max age of Archean	Bedrock	ZCTA/Invariant	U.S. Geological Survey the State Geologic Map Compilation
62	Percent over bedrock with max age of Neoarchean	Bedrock	ZCTA/Invariant	U.S. Geological Survey the State Geologic Map Compilation
63	Percent over bedrock with max age of Proterozoic	Bedrock	ZCTA/Invariant	U.S. Geological Survey the State Geologic Map Compilation
64	Percent over bedrock with max age of Paleoarchean	Bedrock	ZCTA/Invariant	U.S. Geological Survey the State Geologic Map Compilation
65	Percent over bedrock with max age of Mesoarchean	Bedrock	ZCTA/Invariant	U.S. Geological Survey the State Geologic Map Compilation
66	Percent over bedrock with max age of Neoarchean	Bedrock	ZCTA/Invariant	U.S. Geological Survey the State Geologic Map Compilation
67	Percent over bedrock with min age of Paleozoic	Bedrock	ZCTA/Invariant	U.S. Geological Survey the State Geologic Map Compilation
68	Percent over bedrock with min age of Carboniferous	Bedrock	ZCTA/Invariant	U.S. Geological Survey the State Geologic Map Compilation
69	Percent over bedrock with min age of Cambrian	Bedrock	ZCTA/Invariant	U.S. Geological Survey the State Geologic Map Compilation
70	Percent over bedrock with min age of Devonian	Bedrock	ZCTA/Invariant	U.S. Geological Survey the State Geologic Map Compilation
71	Percent over bedrock with min age of Tertiary	Bedrock	ZCTA/Invariant	U.S. Geological Survey the State Geologic Map Compilation
72	Percent over bedrock with min age of Quaternary	Bedrock	ZCTA/Invariant	U.S. Geological Survey the State Geologic Map Compilation
73	Percent over bedrock with min age of Cretaceous	Bedrock	ZCTA/Invariant	U.S. Geological Survey the State Geologic Map Compilation
74	Percent over bedrock with min age of Ordovician	Bedrock	ZCTA/Invariant	U.S. Geological Survey the State Geologic Map Compilation
75	Percent over bedrock with min age of Silurian	Bedrock	ZCTA/Invariant	U.S. Geological Survey the State Geologic Map Compilation

76	Percent over bedrock with min age of Jurassic	Bedrock	ZCTA/Invariant	U.S. Geological Survey the State Geologic Map Compilation
77	Percent over bedrock with min age of Permian	Bedrock	ZCTA/Invariant	U.S. Geological Survey the State Geologic Map Compilation
78	Percent over bedrock with min age of Triassic	Bedrock	ZCTA/Invariant	U.S. Geological Survey the State Geologic Map Compilation
79	Percent over bedrock with min age of Paleoproterozoic	Bedrock	ZCTA/Invariant	U.S. Geological Survey the State Geologic Map Compilation
80	Percent over bedrock with min age of Mesoproterozoic	Bedrock	ZCTA/Invariant	U.S. Geological Survey the State Geologic Map Compilation
81	Percent over bedrock with min age of Neoproterozoic	Bedrock	ZCTA/Invariant	U.S. Geological Survey the State Geologic Map Compilation
82	Percent over bedrock with min age of Mesozoic	Bedrock	ZCTA/Invariant	U.S. Geological Survey the State Geologic Map Compilation
83	Percent over bedrock with min age of PreCambrian	Bedrock	ZCTA/Invariant	U.S. Geological Survey the State Geologic Map Compilation
84	Percent over bedrock with min age of Archean	Bedrock	ZCTA/Invariant	U.S. Geological Survey the State Geologic Map Compilation
85	Percent over bedrock with min age of Phanerozoic	Bedrock	ZCTA/Invariant	U.S. Geological Survey the State Geologic Map Compilation
86	Percent over bedrock with min age of Proterozoic	Bedrock	ZCTA/Invariant	U.S. Geological Survey the State Geologic Map Compilation
87	Percent over bedrock with min age of Neoarchean	Bedrock	ZCTA/Invariant	U.S. Geological Survey the State Geologic Map Compilation
88	Percent over bedrock with min age of Mesoarchean	Bedrock	ZCTA/Invariant	U.S. Geological Survey the State Geologic Map Compilation
89	Percent over bedrock with min age of Paleoarchean	Bedrock	ZCTA/Invariant	U.S. Geological Survey the State Geologic Map Compilation
90	Percent over Sandstone	Bedrock	ZCTA/Invariant	U.S. Geological Survey the State Geologic Map Compilation
91	Percent over Shale	Bedrock	ZCTA/Invariant	U.S. Geological Survey the State Geologic Map Compilation
92	Percent over Limestone	Bedrock	ZCTA/Invariant	U.S. Geological Survey the State Geologic Map Compilation
93	Percent over Sand	Bedrock	ZCTA/Invariant	U.S. Geological Survey the State Geologic Map Compilation
94	Percent over Siltstone	Bedrock	ZCTA/Invariant	U.S. Geological Survey the State Geologic Map Compilation
95	Percent over Coarse -detrital	Bedrock	ZCTA/Invariant	U.S. Geological Survey the State Geologic Map Compilation
96	Percent over Dolostone	Bedrock	ZCTA/Invariant	U.S. Geological Survey the State Geologic Map Compilation
97	Percent over Clay	Bedrock	ZCTA/Invariant	U.S. Geological Survey the State Geologic Map Compilation
98	Percent over Gravel	Bedrock	ZCTA/Invariant	U.S. Geological Survey the State Geologic Map Compilation
99	Percent over Fine -detrital	Bedrock	ZCTA/Invariant	U.S. Geological Survey the State Geologic Map Compilation
100	Percent over Silt	Bedrock	ZCTA/Invariant	U.S. Geological Survey the State Geologic Map Compilation
101	Percent over Basalt	Bedrock	ZCTA/Invariant	U.S. Geological Survey the State Geologic Map Compilation
102	Percent over Conglomerate	Bedrock	ZCTA/Invariant	U.S. Geological Survey the State Geologic Map Compilation

103	Percent over Mudstone	Bedrock	ZCTA/Invariant	U.S. Geological Survey the State Geologic Map Compilation
104	Percent over Andesite	Bedrock	ZCTA/Invariant	U.S. Geological Survey the State Geologic Map Compilation
105	Percent over Unconsolidated	Bedrock	ZCTA/Invariant	U.S. Geological Survey the State Geologic Map Compilation
106	Percent over Rhyolite	Bedrock	ZCTA/Invariant	U.S. Geological Survey the State Geologic Map Compilation
107	Percent over Quartzite	Bedrock	ZCTA/Invariant	U.S. Geological Survey the State Geologic Map Compilation
108	Percent over Dacite	Bedrock	ZCTA/Invariant	U.S. Geological Survey the State Geologic Map Compilation
109	Percent over Gneiss	Bedrock	ZCTA/Invariant	U.S. Geological Survey the State Geologic Map Compilation
110	Percent over Claystone	Bedrock	ZCTA/Invariant	U.S. Geological Survey the State Geologic Map Compilation
111	Percent over Volcanic	Bedrock	ZCTA/Invariant	U.S. Geological Survey the State Geologic Map Compilation
112	Percent over Granite	Bedrock	ZCTA/Invariant	U.S. Geological Survey the State Geologic Map Compilation
113	Percent over Felsic-volcanic	Bedrock	ZCTA/Invariant	U.S. Geological Survey the State Geologic Map Compilation
114	Soil available water capacity	Soil	9 km/Invariant	U.S. Department of Agriculture (STATSGO)
115	Soil organic matter	Soil	9 km/Invariant	U.S. Department of Agriculture (STATSGO)
116	Soil saturated hydraulic conductivity	Soil	9 km/Invariant	U.S. Department of Agriculture (STATSGO)
117	Soil vertical permeability (KV)	Soil	9 km/Invariant	U.S. Department of Agriculture (STATSGO)
118	Soil bulk density	Soil	9 km/Invariant	U.S. Department of Agriculture (STATSGO)
119	Soil field capacity	Soil	9 km/Invariant	U.S. Department of Agriculture (STATSGO)
120	Percent of gravel in soil	Soil	9 km/Invariant	U.S. Department of Agriculture (STATSGO)
121	Percent of coarse sand in soil	Soil	9 km/Invariant	U.S. Department of Agriculture (STATSGO)
122	Percent of fine sand in soil	Soil	9 km/Invariant	U.S. Department of Agriculture (STATSGO)
123	Soil porosity	Soil	9 km/Invariant	U.S. Department of Agriculture (STATSGO)
124	Soil erodibility (K factor)	Soil	9 km/Invariant	U.S. Department of Agriculture (STATSGO)
125	Soil thickness	Soil	9 km/Invariant	U.S. Department of Agriculture (STATSGO)
126	Number of housing units	Architectural	ZCTA/Invariant	American Community Survey
127	Number of single-family units	Architectural	ZCTA/Invariant	American Community Survey
128	Number of two-unit housing	Architectural	ZCTA/Invariant	American Community Survey
129	Number of three to four-unit housing	Architectural	ZCTA/Invariant	American Community Survey
130	Number of five to nine-unit housing	Architectural	ZCTA/Invariant	American Community Survey

131	Number of ten to nineteen-unit housing	Architectural	ZCTA/Invariant	American Community Survey
132	Number of over twenty-unit housing	Architectural	ZCTA/Invariant	American Community Survey
133	Number of over fifty-unit housing	Architectural	ZCTA/Invariant	American Community Survey
134	Number of units constructed before 1939	Architectural	ZCTA/Invariant	American Community Survey
135	Number of units constructed in 1940-1949	Architectural	ZCTA/Invariant	American Community Survey
136	Number of units constructed in 1950-1959	Architectural	ZCTA/Invariant	American Community Survey
137	Number of units constructed in 1960-1969	Architectural	ZCTA/Invariant	American Community Survey
138	Number of units constructed in 1970-1979	Architectural	ZCTA/Invariant	American Community Survey
139	Number of units constructed in 1980-1989	Architectural	ZCTA/Invariant	American Community Survey
140	Number of units constructed in 1990-1999	Architectural	ZCTA/Invariant	American Community Survey
141	Number of units constructed in 2000-2009	Architectural	ZCTA/Invariant	American Community Survey
142	Number of units constructed in 2010-2013	Architectural	ZCTA/Invariant	American Community Survey
143	Number of units constructed after 2014	Architectural	ZCTA/Invariant	American Community Survey
144	Number of one-room units	Architectural	ZCTA/Invariant	American Community Survey
145	Number of two-room units	Architectural	ZCTA/Invariant	American Community Survey
146	Number of three-room units	Architectural	ZCTA/Invariant	American Community Survey
147	Number of four-room units	Architectural	ZCTA/Invariant	American Community Survey
148	Number of five-room units	Architectural	ZCTA/Invariant	American Community Survey
149	Number of six-room units	Architectural	ZCTA/Invariant	American Community Survey
150	Number of seven-room units	Architectural	ZCTA/Invariant	American Community Survey
151	Number of eight-room units	Architectural	ZCTA/Invariant	American Community Survey
152	Number of over nine-room units	Architectural	ZCTA/Invariant	American Community Survey
153	Number of units with no bedroom	Architectural	ZCTA/Invariant	American Community Survey
154	Number of one-bedroom units	Architectural	ZCTA/Invariant	American Community Survey
155	Number of two-bedroom units	Architectural	ZCTA/Invariant	American Community Survey
156	Number of three-bedroom units	Architectural	ZCTA/Invariant	American Community Survey
157	Number of four-bedroom units	Architectural	ZCTA/Invariant	American Community Survey
158	Number of over five-bedroom units	Architectural	ZCTA/Invariant	American Community Survey
159	Number of units fueled by natural gas	Architectural	ZCTA/Invariant	American Community Survey
160	Number of units fueled by oil	Architectural	ZCTA/Invariant	American Community Survey
161	Number of units fueled by electricity	Architectural	ZCTA/Invariant	American Community Survey

162	Number of units fueled by coal	Architectural	ZCTA/Invariant	American Community Survey
163	Number of units fueled by Solar	Architectural	ZCTA/Invariant	American Community Survey
164	Number of units without heating fuel	Architectural	ZCTA/Invariant	American Community Survey
165	Population density	Socioeconomic	ZCTA/Invariant	U.S. Census 2015
166	Median property value	Socioeconomic	ZCTA/Invariant	U.S. Census 2015
167	Mean household income	Socioeconomic	ZCTA/Invariant	U.S. Census 2015
168	Percent of property occupied by owner	Socioeconomic	ZCTA/Invariant	U.S. Census 2015
169	Relative humidity	Meteorological	32 km/Daily	North America Regional Reanalysis Data
170	Soil temperature	Meteorological	32 km/Daily	North America Regional Reanalysis Data
171	Soil moisture content	Meteorological	32 km/Daily	North America Regional Reanalysis Data
172	Snow cover	Meteorological	32 km/Daily	North America Regional Reanalysis Data
173	Volumetric soil moisture (frozen + liquid) fraction	Meteorological	32 km/Daily	North America Regional Reanalysis Data
174	Accumulated precipitation	Meteorological	32 km/Daily	North America Regional Reanalysis Data
175	Barometric pressure	Meteorological	32 km/Daily	North America Regional Reanalysis Data
176	U-wind	Meteorological	32 km/Daily	United States Environmental Protection Agency
177	V-wind	Meteorological	32 km/Daily	United States Environmental Protection Agency
178	Air temperature	Meteorological	32 km/Daily	United States Environmental Protection Agency
179	Albeno	Meteorological	32 km/Daily	United States Environmental Protection Agency
180	Planetary boundary layer height	Meteorological	32 km/Daily	United States Environmental Protection Agency
181	Gross beta radiation	Meteorological	Monitor/Daily	United States Environmental Protection Agency
182	Year	Temporal Trend		
183	Month of year	Temporal Trend		
184	Latitude	Spatial trend		
185	Longitude	Spatial trend		
186	Radon disclosure requirement	Policy-related	State/Yearly	State radon laws and program materials
187	Radon test program	Policy-related	State/Yearly	State radon laws and program materials
188	Measurement requirement	Policy-related	State/Yearly	State radon laws and program materials
189	Housing affordability	Policy-related	County/Yearly	Federal Reserve Bank of Atlanta

Table S2. Population-weighted percentages and numbers of residents exceeding selected indoor radon thresholds derived from different training models

	Region	>74 Bq/m³ (2 pCi/L)		>148 Bq/m³ (4 pCi/L)		>370 Bq/m³ (10 pCi/L)	
		Percentage (%)	Number (million)	Percentage (%)	Number (million)	Percentage (%)	Number (million)
Main model	National	40.5	121.4	14.3	42.8	2.1	6.2
	Midwest	60.6	40.5	27.9	18.7	4.7	3.1
	Northeast	44.5	23.9	16.7	9.0	2.7	1.5
	West	34.5	22.5	8.8	5.8	0.9	0.6
	South	30.3	34.5	8.2	9.4	0.9	1.0
Benchmark model	National	39.5	118.3	14.4	43.1	2.3	6.7
	Midwest	59.8	40.0	28.0	18.7	5.0	3.3
	Northeast	43.6	23.4	16.7	9.0	2.9	1.6
	West	32.6	21.2	8.8	5.7	1.0	0.7
	South	29.5	33.6	8.5	9.7	1.0	1.2
Parameter-based model	National	41.4	123.9	17.0	51.1	3.2	9.6
	Midwest	56.8	37.9	28.4	19.0	6.4	4.3
	Northeast	43.9	23.6	19.3	10.4	3.9	2.1
	West	36.9	24.1	12.8	8.4	1.9	1.2
	South	33.6	38.3	11.7	13.3	1.7	2.0
Uncertainty-weighted model	National	35.3	105.9	10.0	30.0	0.9	2.8
	Midwest	53.1	35.5	20.2	13.5	2.2	1.5
	Northeast	36.3	19.6	10.6	5.7	1.1	0.6
	West	30.0	19.6	6.3	4.1	0.4	0.3
	South	27.4	31.2	5.9	6.7	0.4	0.4

Table S3. Population-weighted threshold exceedance probabilities and bootstrap standard-error-based 95% CI by region.

	>74 Bq/m³ (2 pCi/L)		>148 Bq/m³ (4 pCi/L)		>370 Bq/m³ (10 pCi/L)	
	Percentage, %	95% CI, %	Percentage, %	95% CI, %	Percentage, %	95% CI, %
National	40.53	(40.48, 40.58)	14.28	(14.26, 14.31)	2.07	(2.06, 2.08)
Midwest	60.56	(60.51, 60.62)	27.95	(27.90, 27.99)	4.68	(4.66, 4.69)
Northeast	44.47	(44.41, 44.52)	16.74	(16.71, 16.76)	2.73	(2.72, 2.74)
West	34.52	(34.46, 34.58)	8.83	(8.80, 8.86)	0.90	(0.89, 0.90)
South	30.32	(30.27, 30.37)	8.21	(8.19, 8.24)	0.90	(0.90, 0.91)

Note: The bootstrap standard-error-based 95% CI were calculated using the bootstrap standard error, defined as the standard deviation of the bootstrap estimates.

Table S4. MAE (Log-transformed) for the 10th, 25th, 50th, 75th, and 90th percentiles of radon concentrations based on 10-fold CV derived from different training models.

	Log scale MAE				
	P10	P25	P50	P75	P90
Main model	0.319	0.237	0.204	0.203	0.234
Benchmark model	0.403	0.337	0.298	0.289	0.326
Uncertainty-weighted model	0.304	0.232	0.210	0.228	0.307

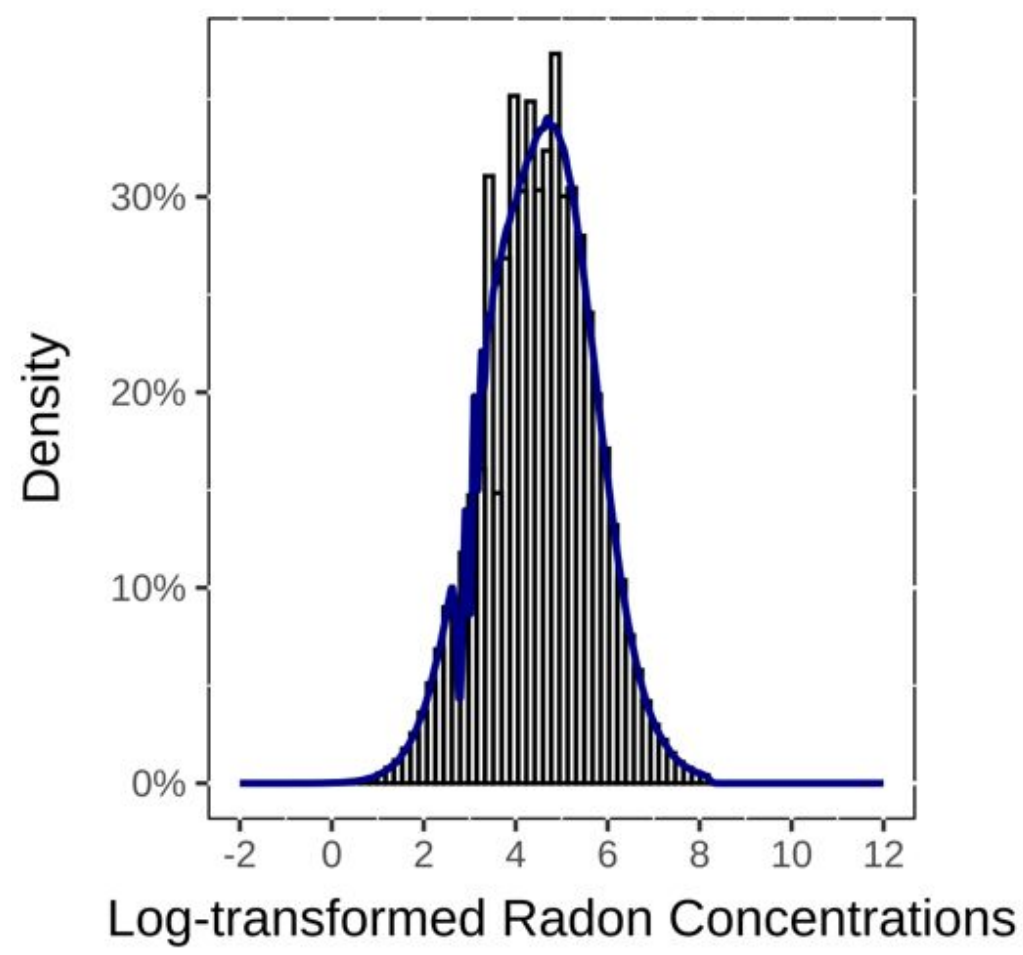

Fig. S1. Density distribution of national log-transformed indoor radon measurements.

Note: The blue line represents the smoothed density curve.

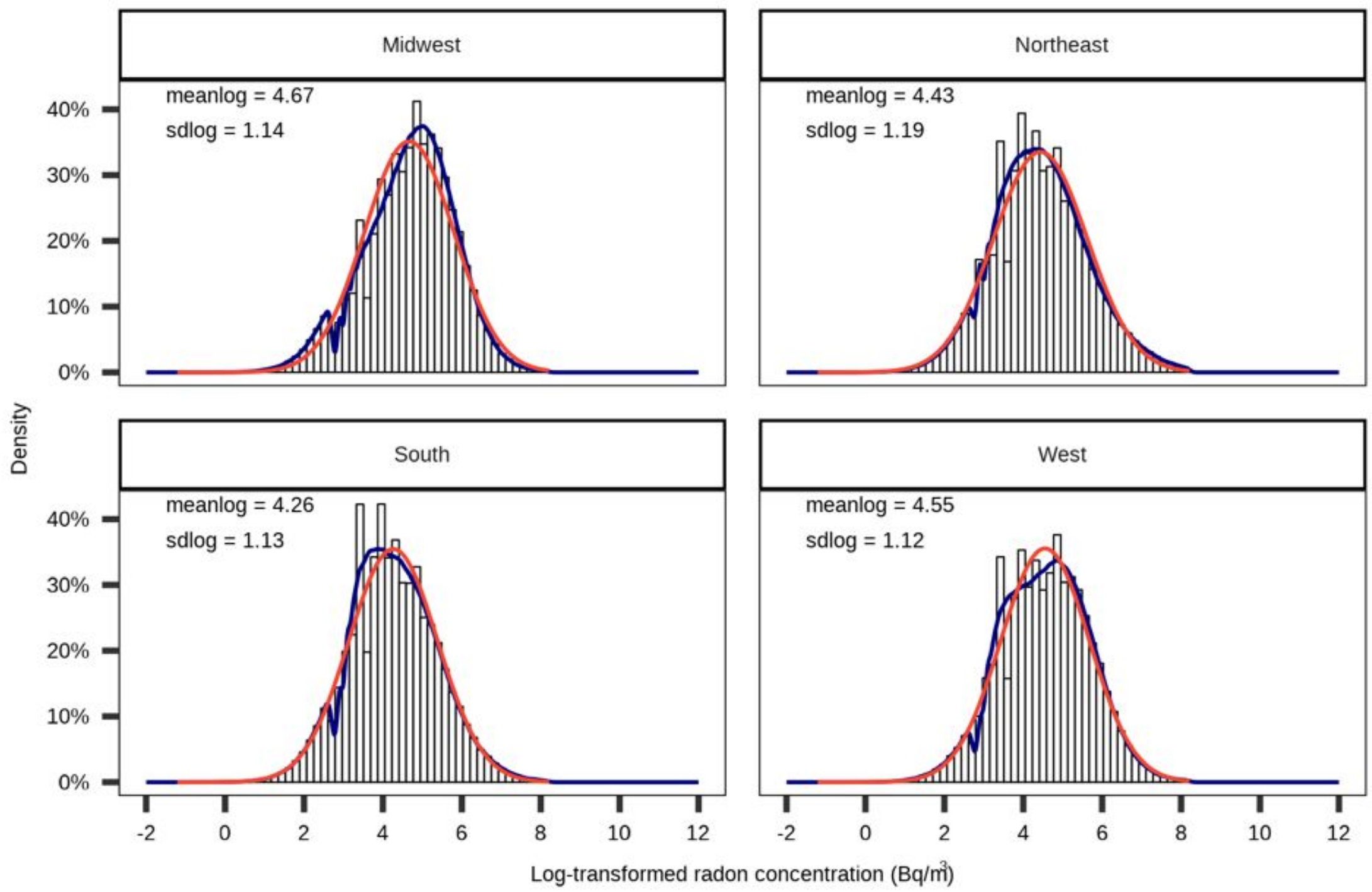

Fig. S2. Regional distributions of log-transformed indoor radon measurements.

Note: The blue line represents the smoothed density curve of measured indoor radon concentrations. The red line represents the fitted normal density curve.

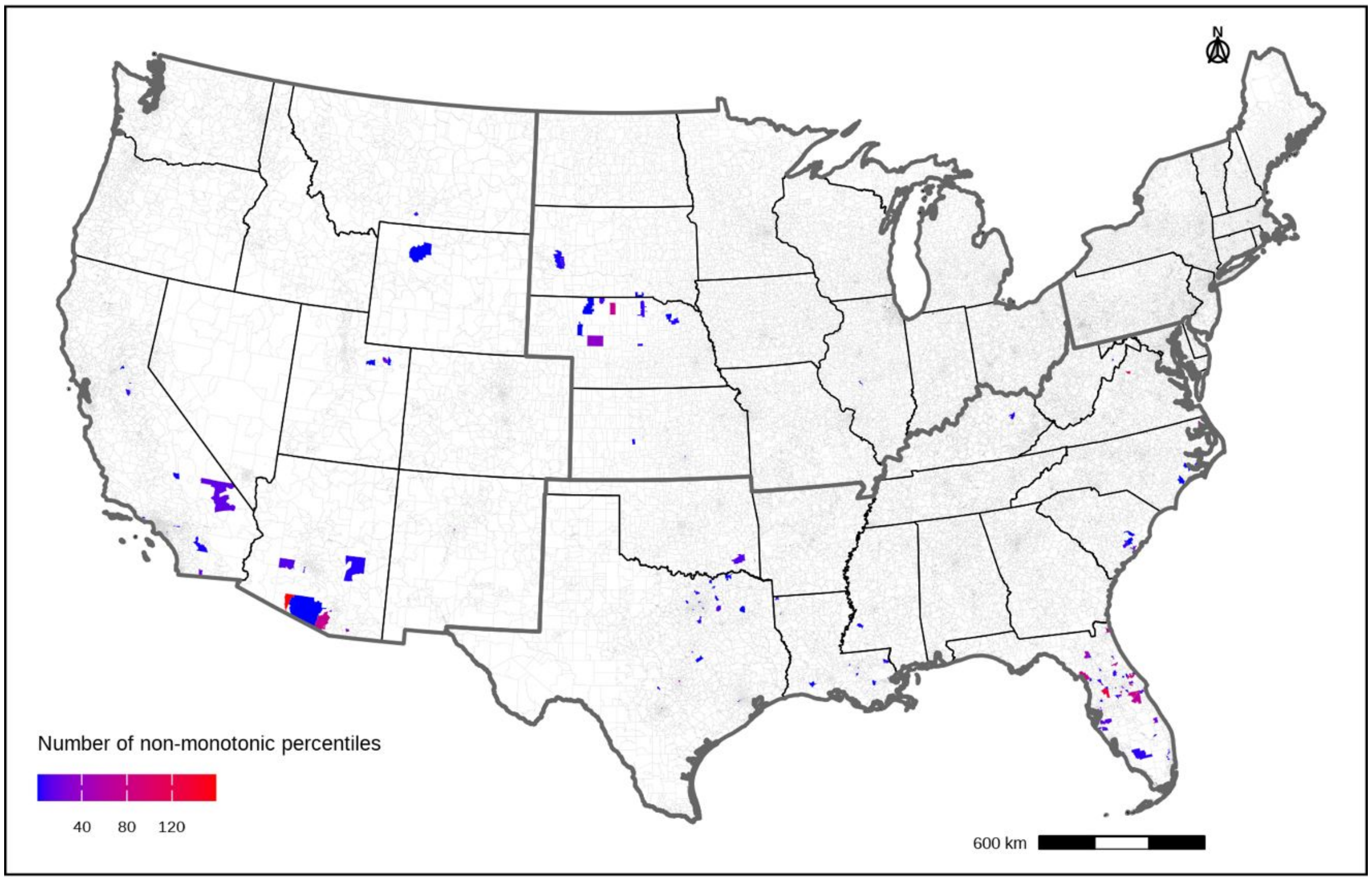

Fig. S3. Spatial distribution of ZCTAs with non-monotonic predicted radon percentiles.

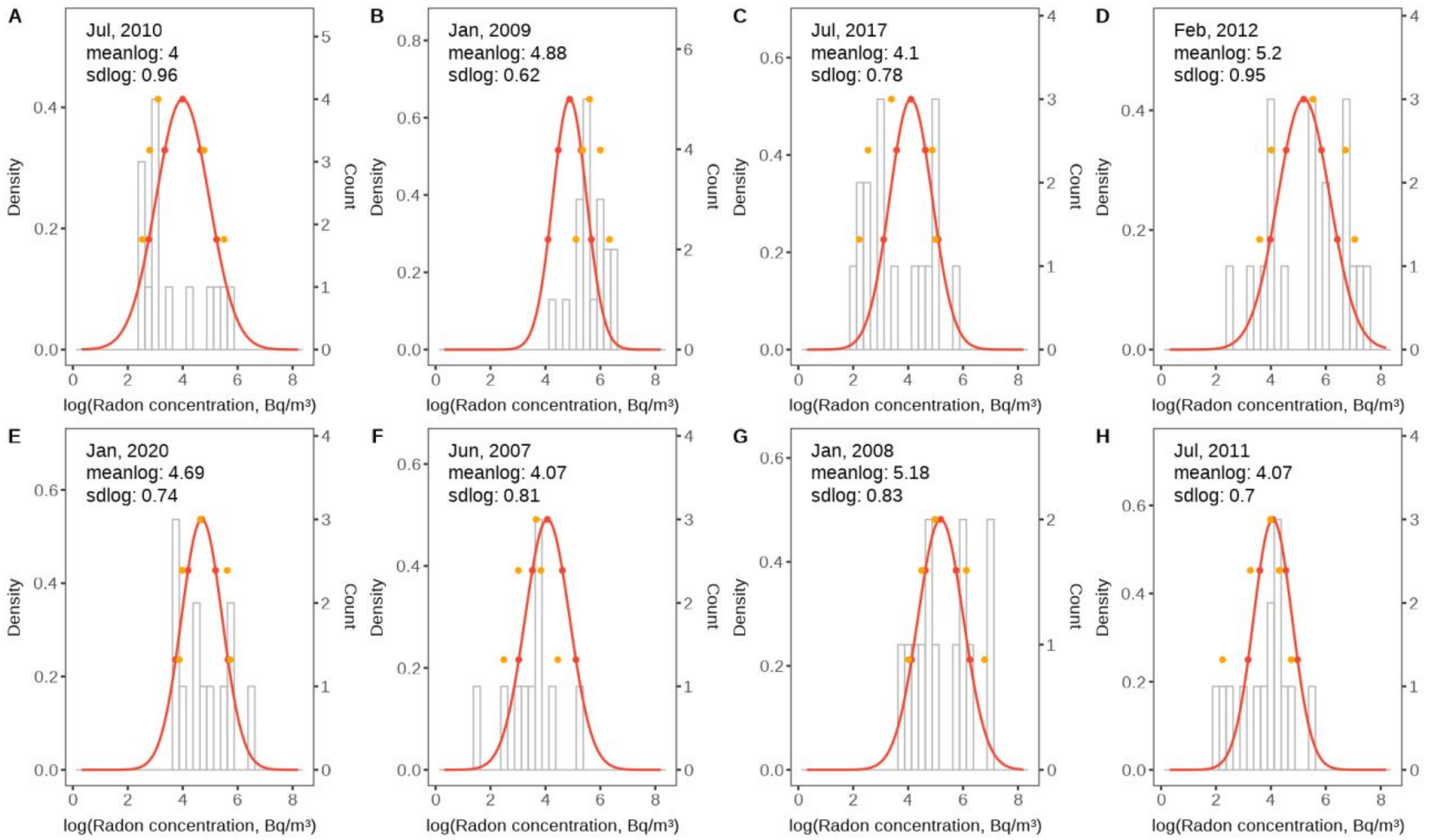

Fig. S4. Comparisons between fitted and measured ZCTA-level indoor radon concentrations on log scale.

Note: Panels A–H show the distributions of indoor radon concentrations for eight illustrative locations (selected across regions and concentration settings; not restricted to the same year): A, Cedar Falls, Iowa; B, Green Bay, Wisconsin; C, Sharon, Massachusetts; D, York, Pennsylvania; E, Bonita Springs, Florida; F, Madison, Alabama; G, Albuquerque, New Mexico; and H, Salt Lake City, Utah.
The red curve represents the normal density implied by the lognormal distribution fitted to the predicted radon percentiles for each selected ZCTA. Red points indicate the corresponding percentiles derived from the fitted distribution, while orange points denote the 10th, 25th, 50th, 75th, and 90th percentiles derived from the measured radon concentrations. Histograms represent the distribution of radon measurements after log transformation.

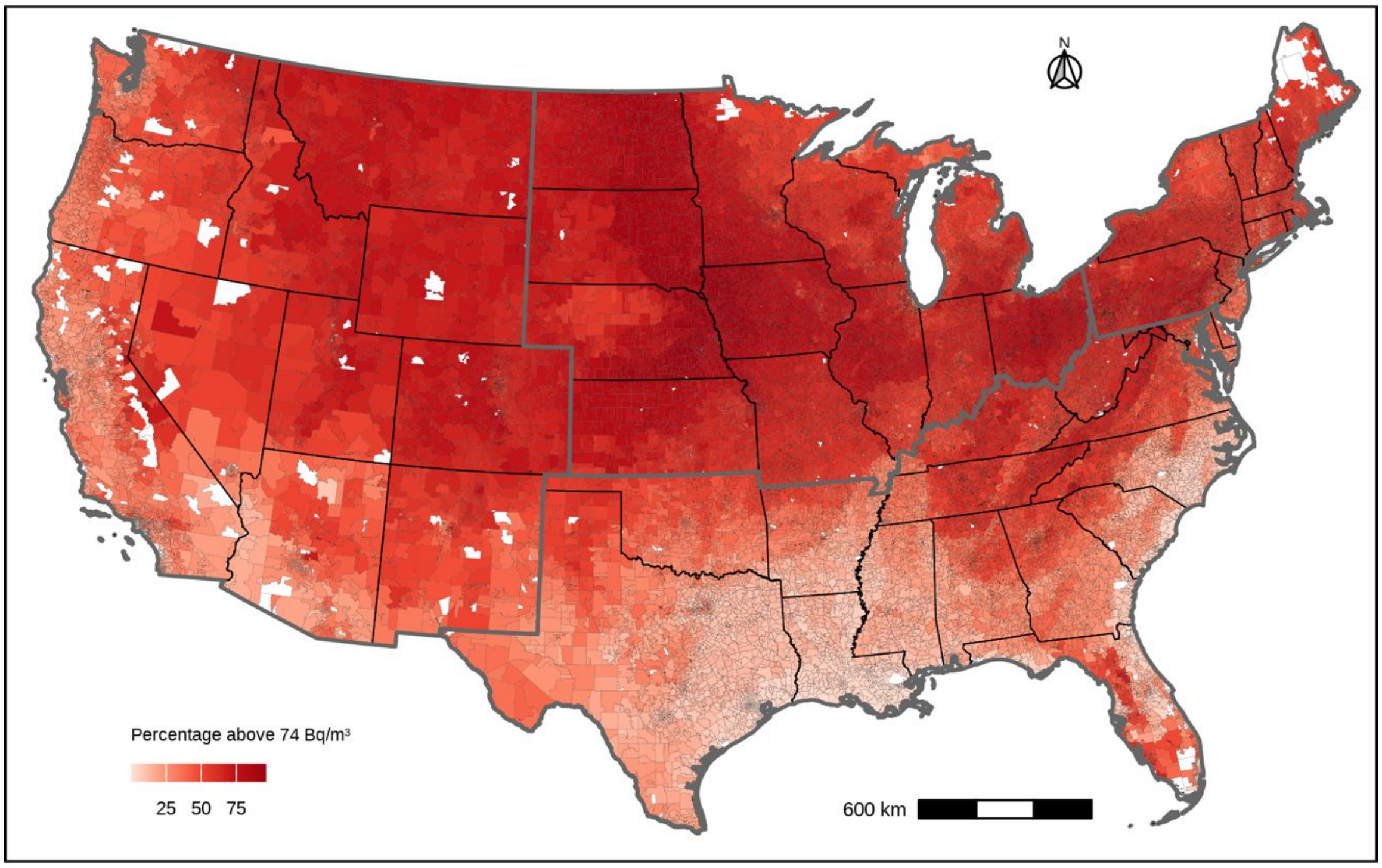

Fig. S5. Percentage of predicted ZCTA-level indoor radon concentrations exceeding 74 Bq/m³ at the screening floor across the United States.

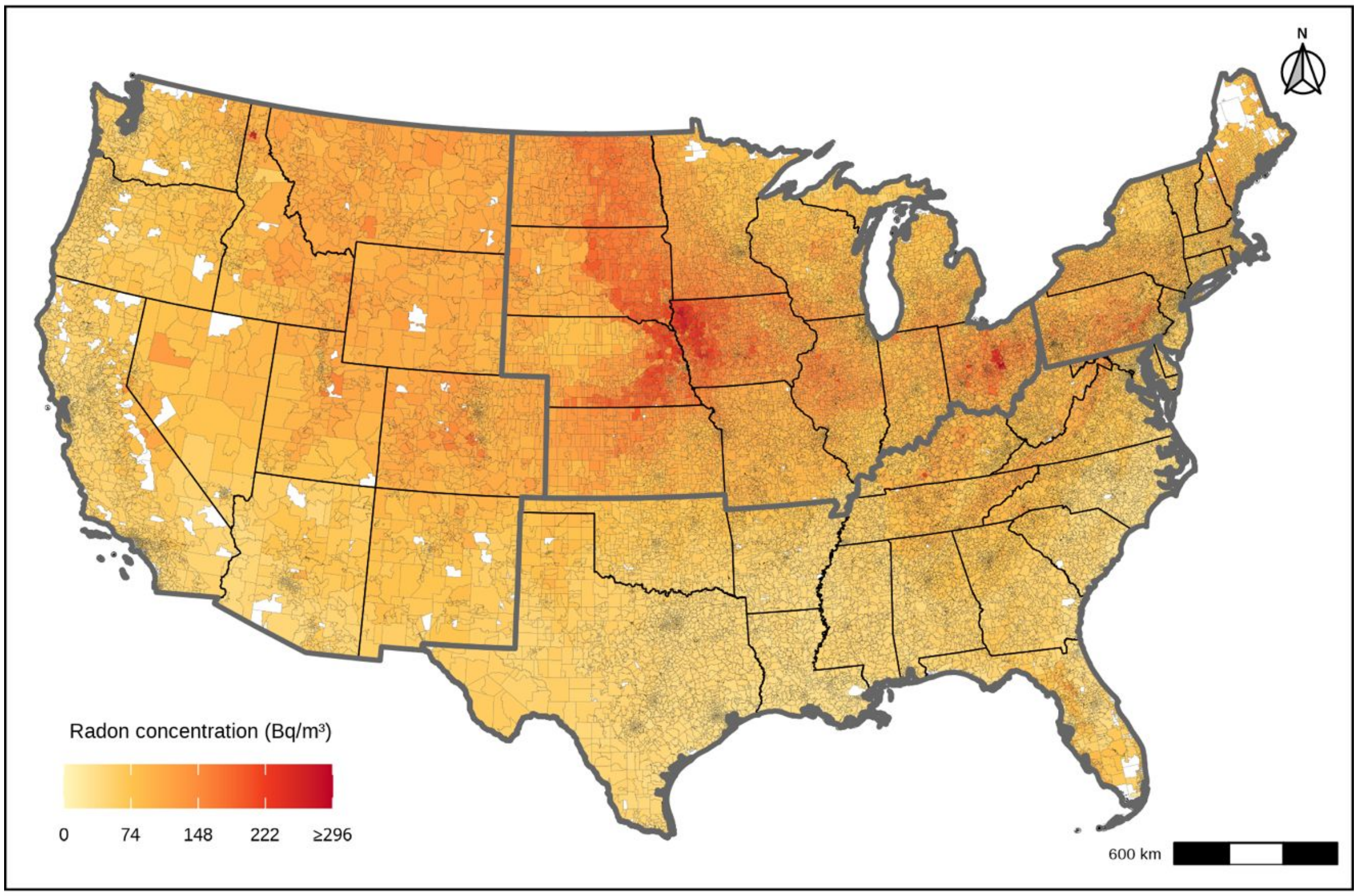

Fig. S6. The median of average predicted ZCTA-level radon concentrations at the screening floor from 2000 to 2021.

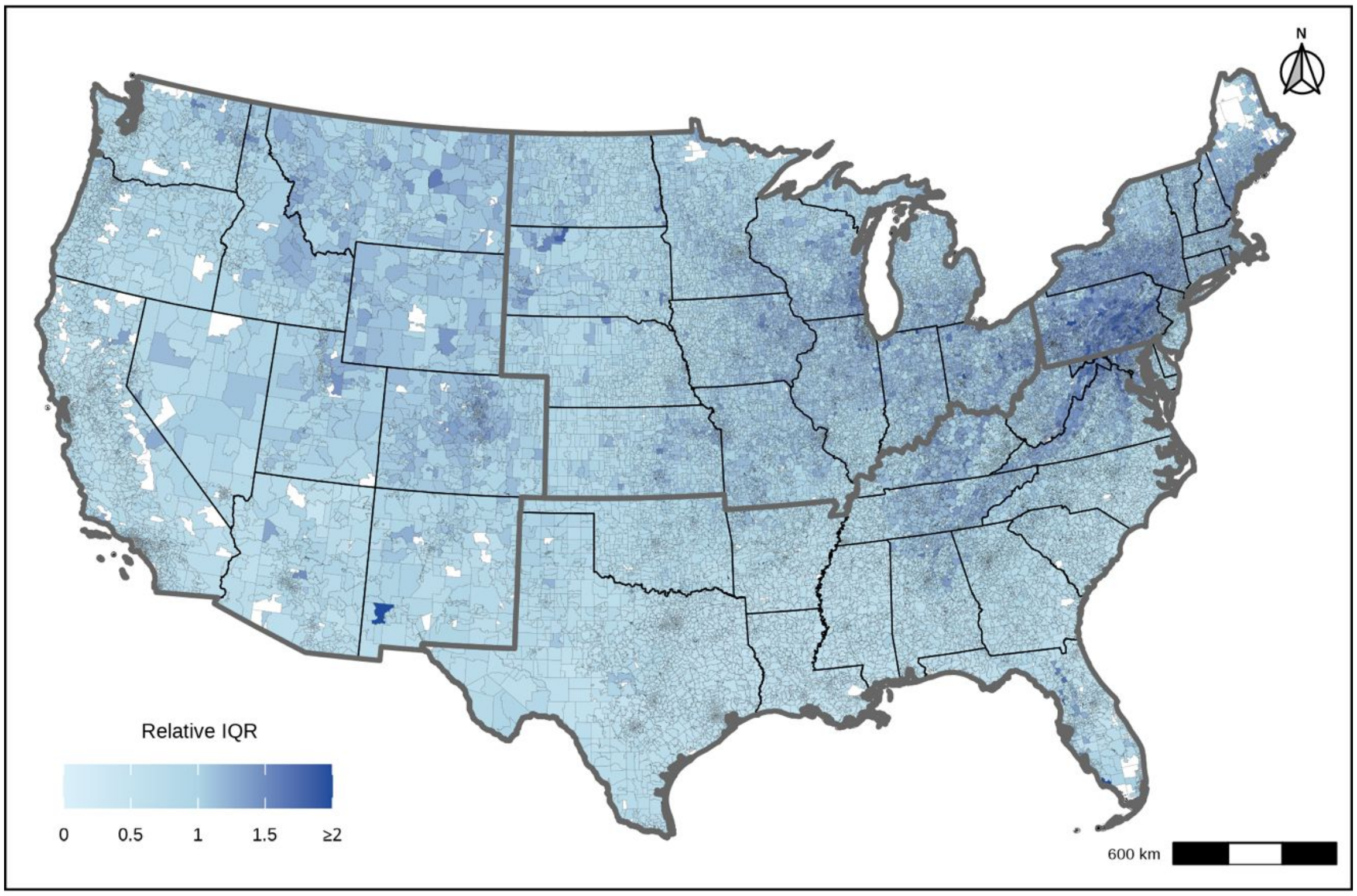

Fig. S7. The relative interquartile range (IQR) of average predicted ZCTA-level radon concentrations at the screening floor from 2000 to 2021.

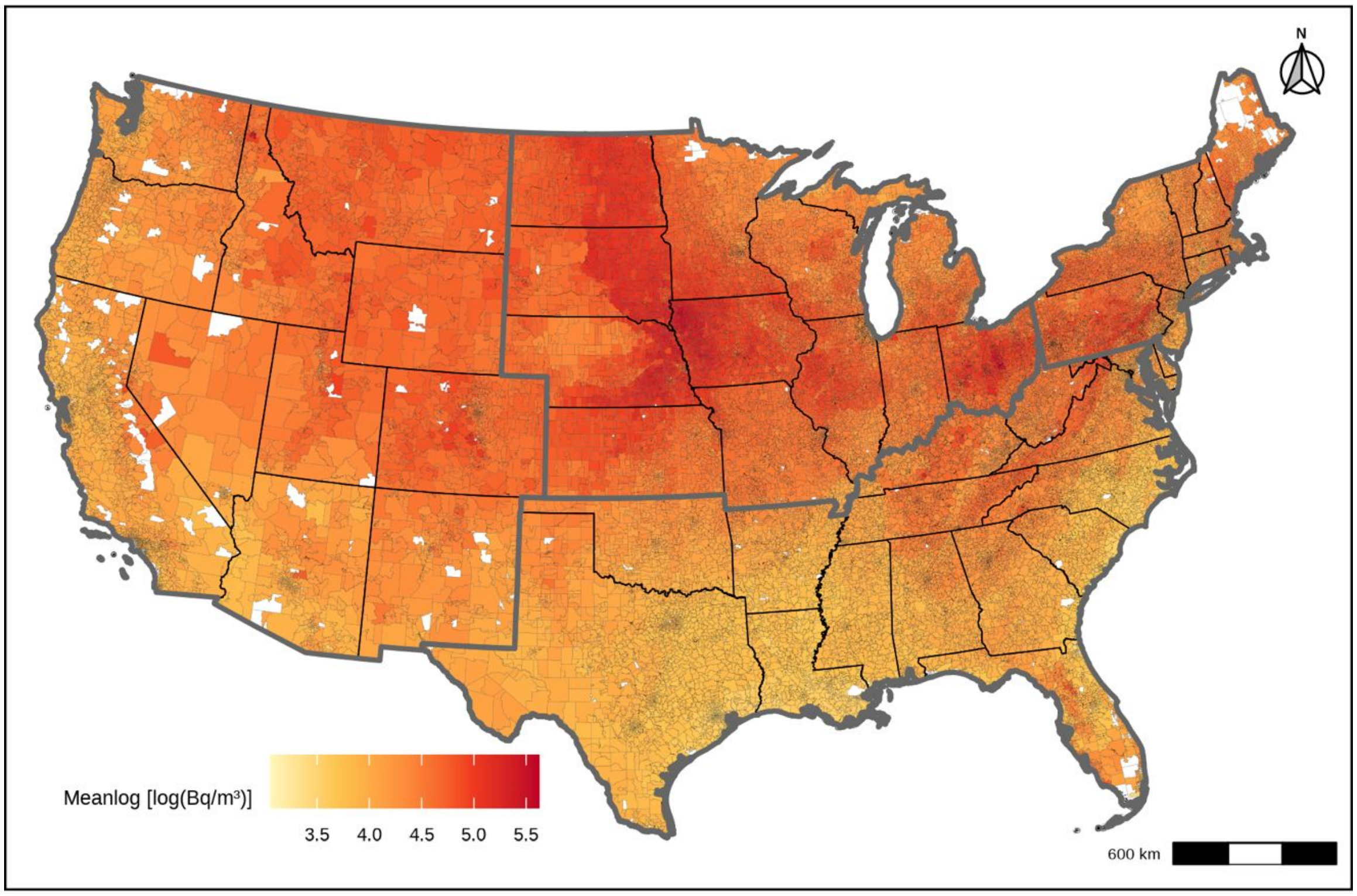

Fig. S8. Spatial distribution of the long-term mean of log-transformed predicted radon concentration (meanlog) at the screening floor averaged over 2000–2021.

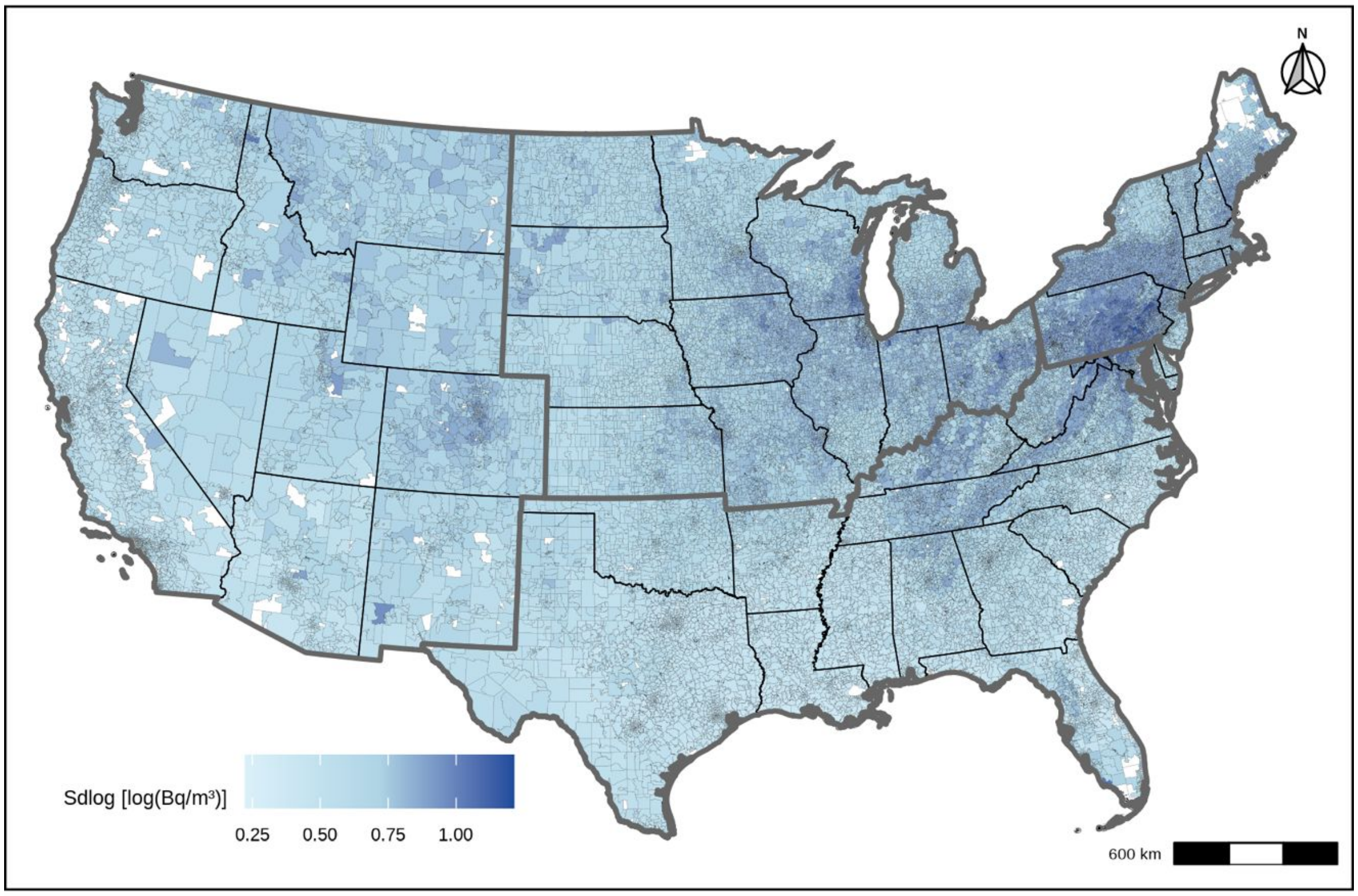

Fig. S9. Spatial distribution of the long-term standard deviation of log-transformed predicted radon concentration (sdlog) at the screening floor averaged over 2000–2021.

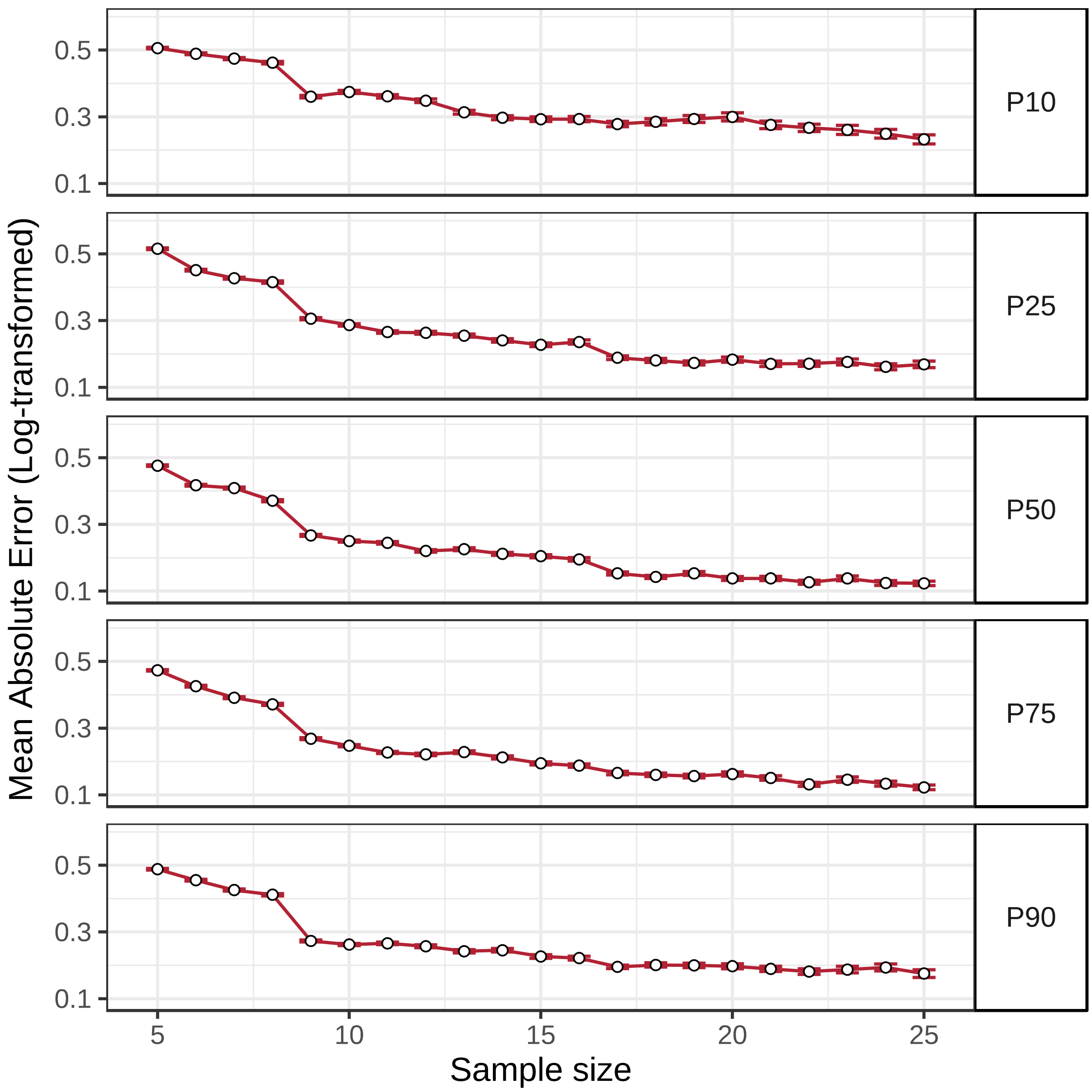

Fig. S10. Trend of MAE (Log-transformed) against sample size for the 10th, 25th, 50th, 75th, and 90th percentiles of radon concentrations based on 10-fold CV.

Note: Error bars represent 95% confidence intervals for the mean absolute error (log-transformed scale).

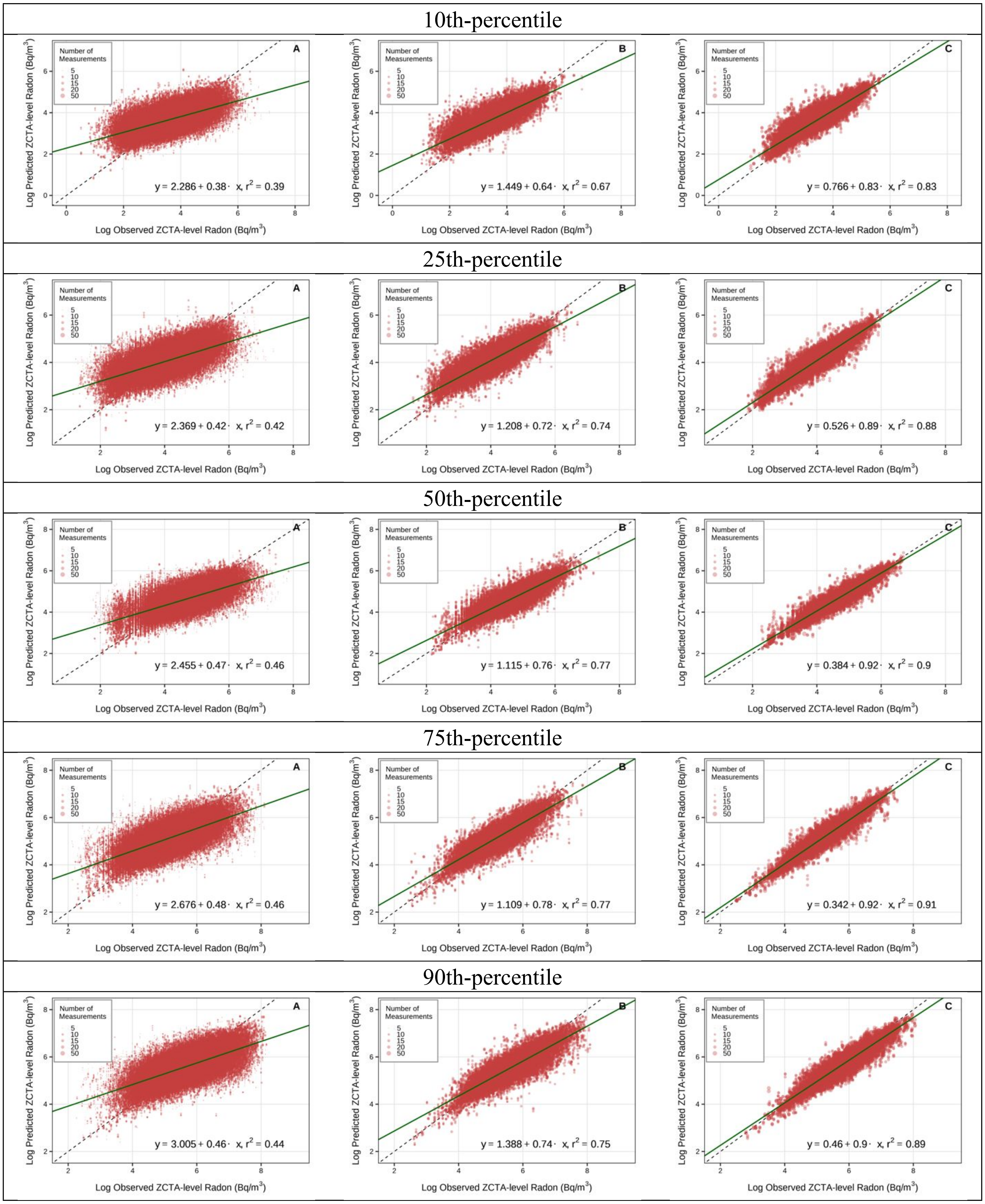

Fig. S11. The scatter plots between the predicted and observed radon concentrations by percentiles, stratified by the sample size of observation, according to the results of 10-fold cross validation.

Note: Panel A for ZCTA-level observations with sample size from 5 to 10; Panel B for ZCTA -level observations with sample size from 10 to 15; Panel C for ZCTA -level observations with sample size over 15; The legend number of measurements represents the number of indoor radon measurements used to calculate the observed ZCTA-level radon percentile, with larger points indicating estimates based on more measurements. The green solid line indicates the fitted linear regression line, and the black dashed line indicates the 1:1 reference line.

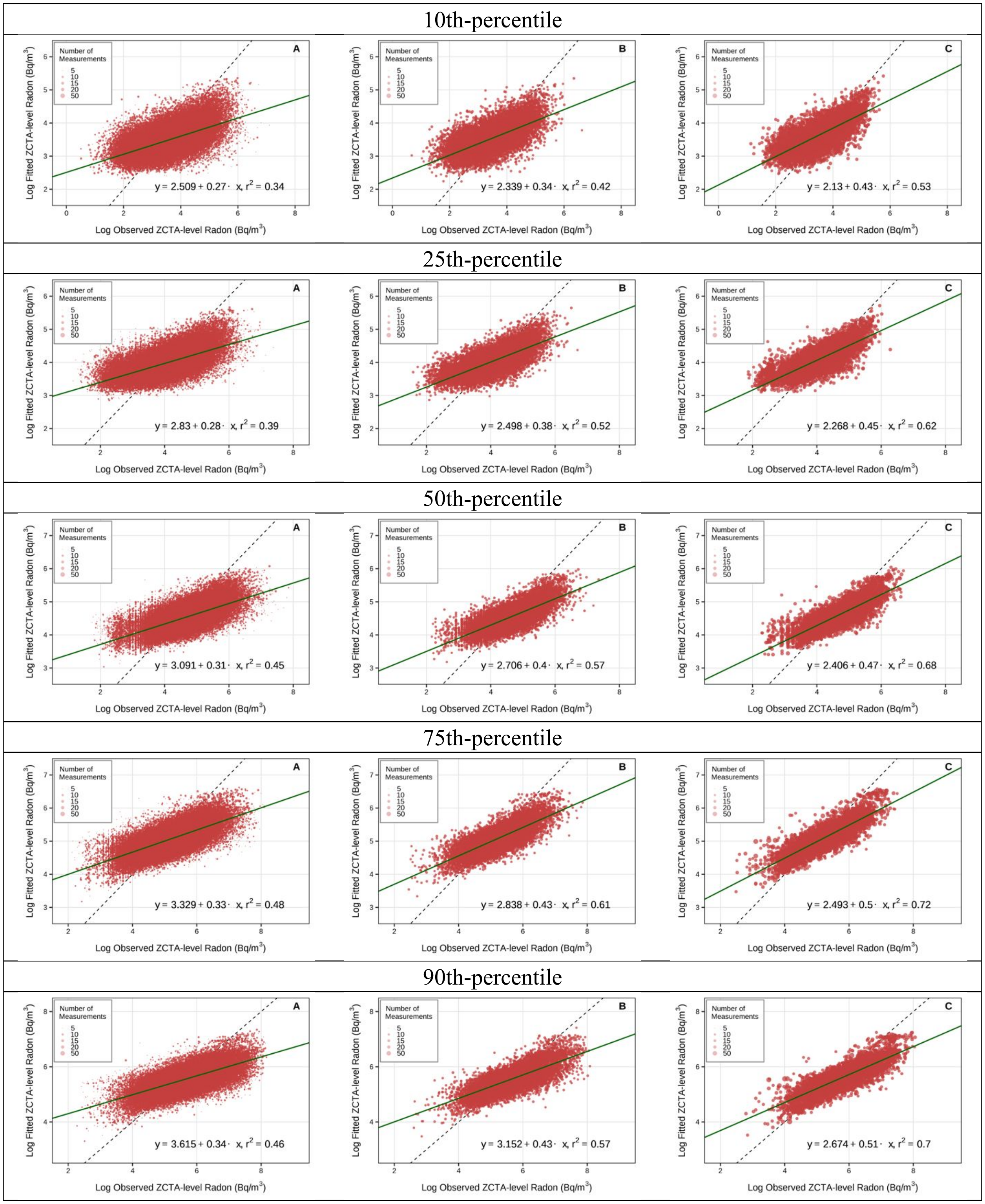

Fig. S12. The scatter plots between the fitted and observed radon concentrations by percentiles, stratified by the sample size of observation.

Note: Panel A for ZCTA-level observations with sample size from 5 to 10; Panel B for ZCTA -level observations with sample size from 10 to 15; Panel C for ZCTA -level observations with sample size over 15; The legend number of measurements represents the number of indoor radon measurements used to calculate the observed ZCTA-level radon percentile, with larger points indicating estimates based on more measurements. The green solid line indicates the fitted linear regression line, and the black dashed line indicates the 1:1 reference line.

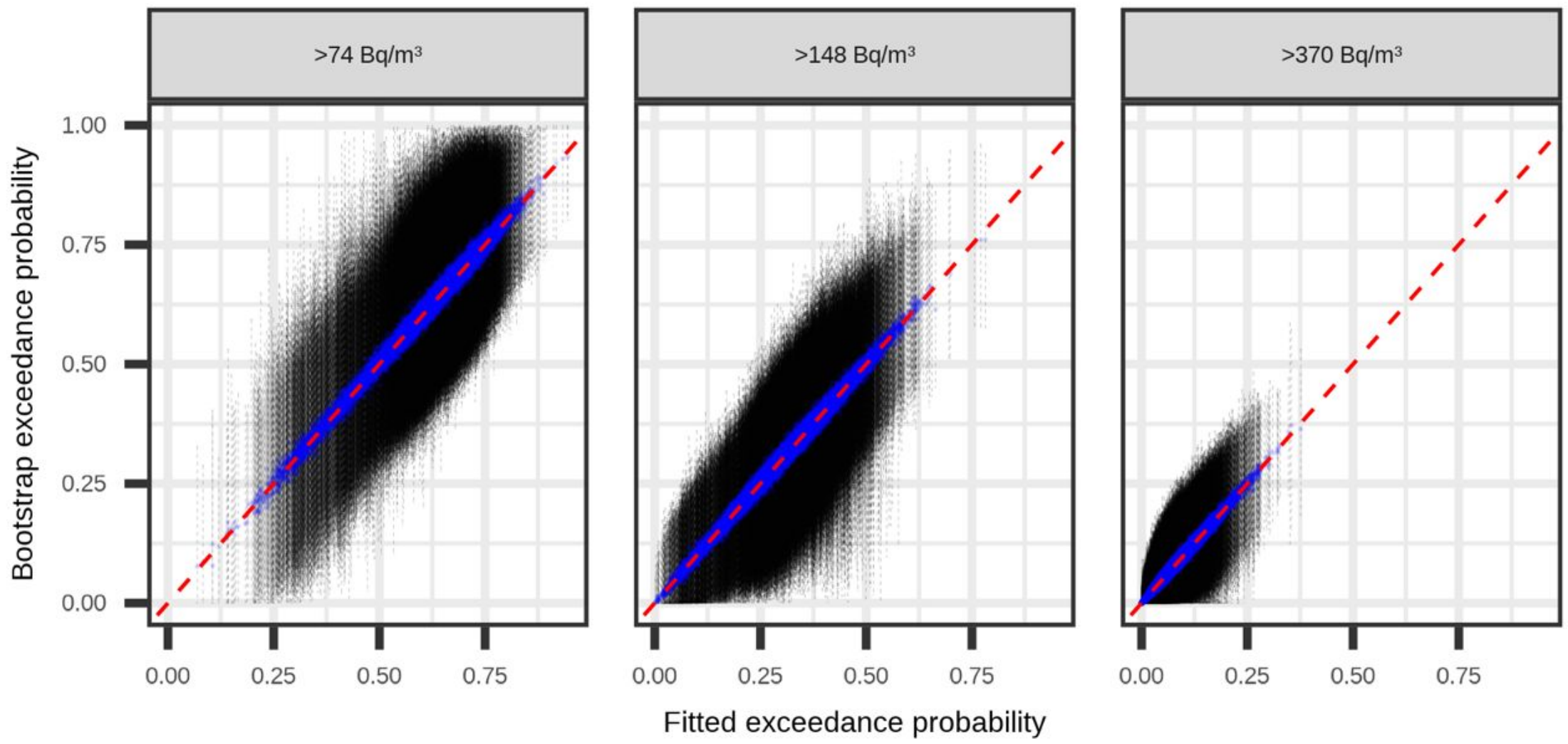

Fig. S13. Comparison of bootstrap-based and fitted exceedance probabilities in Pennsylvania in 2015 across different thresholds.

Note: The red dashed line represents the 1:1 reference line. The blue points represent the median bootstrap exceedance probabilities, and the black vertical segments represent the corresponding 95% bootstrap confidence intervals.

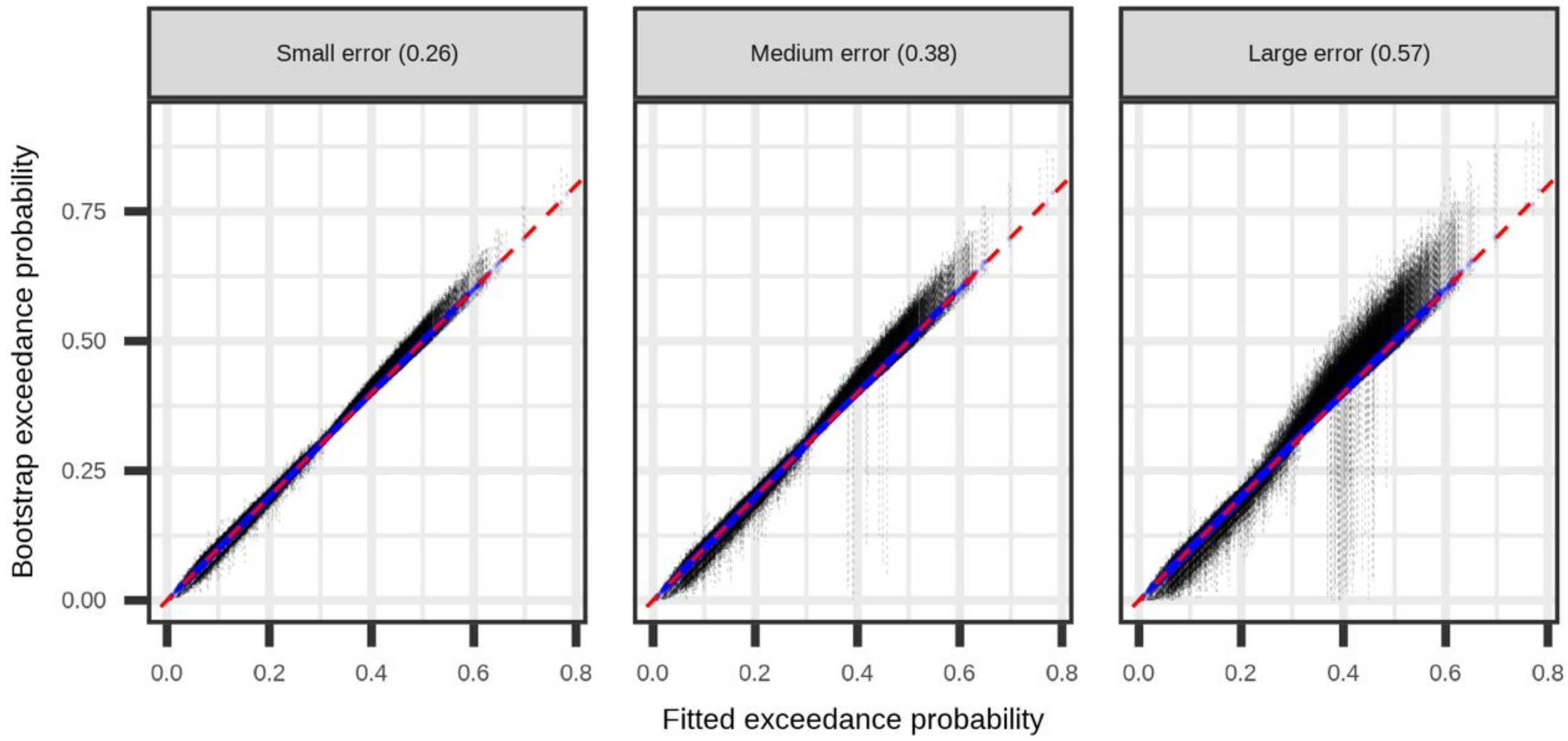

Fig. S14. Lower-tail uncertainty analysis for exceedance probabilities above 148 Bq/m³ in Pennsylvania in 2015 across different errors.

Note: The red dashed line represents the 1:1 reference line. The blue points represent the median bootstrap exceedance probabilities, and the black vertical segments represent the corresponding 95% bootstrap confidence intervals.

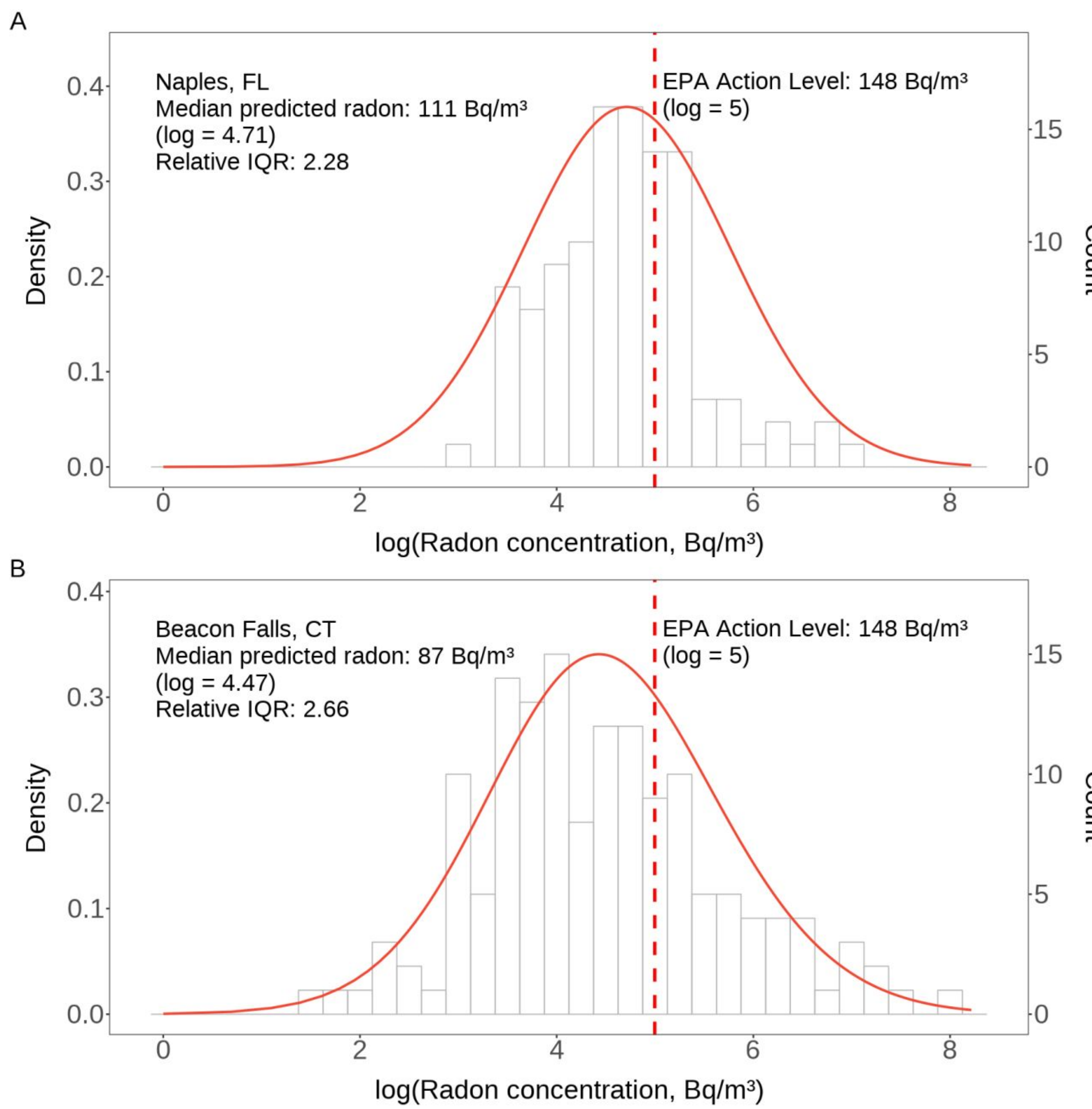

Fig. S15. Measured and fitted distributions of indoor radon concentrations illustrating within-ZCTA exposure heterogeneity during 2000-2021 in selected ZCTAs.

Note: The red curve represents the normal density implied by the lognormal distribution fitted to the predicted radon percentiles for each selected ZCTA. Histograms represent measured radon concentrations after log transformation.

References

1. American Association of Radon Scientists and Technologists. Radon Measurement Systems Quality Assurance: ANSI/AARST MS-QA-2019. Hendersonville, NC: American Association of Radon Scientists and Technologists; 2019.
2. Sun K, Majdan M, Field DW, Field RW. Field comparison of commercially available short-term radon detectors. *Health physics* 2006; **91**(3): 221–6.
3. Sun K, Budd G, McLemore S, Field RW. Blind testing of commercially available short-term radon detectors. *Health physics* 2008; **94**(6): 548–57.
4. Hahn EJ, Gokun Y, Andrews WM, Jr., et al. Radon potential, geologic formations, and lung cancer risk. *Prev Med Rep* 2015; **2**: 342–6.
5. Gundersen LC, Schumann RR. Mapping the radon potential of the United States: examples from the Appalachians. *Environment International* 1996; **22**: 829–37.
6. Gesell TF, Lowder WM. Natural radiation environment III: Department of Energy, Oak Ridge, TN (USA). Technical Information Center, 1980.
7. Sabbarese C, Ambrosino F, D'Onofrio A. Development of radon transport model in different types of dwellings to assess indoor activity concentration. *J Environ Radioact* 2021; **227**: 106501.
8. Stanley FKT, Irvine JL, Jacques WR, et al. Radon exposure is rising steadily within the modern North American residential environment, and is increasingly uniform across seasons. *Sci Rep* 2019; **9**(1): 18472.
9. Şen G, Içhedef M, Saç M, Yener G. Effect of natural gas usage on indoor radon levels. *Journal of Radioanalytical and Nuclear Chemistry* 2013; **295**(1): 277–82.
10. Gelman A, Krantz DH, Lin C, Price PN. Analysis of local decisions using hierarchical modeling, applied to home radon measurement and remediation. *Statistical Science* 1999; **14**(3): 305–37.
11. Schubert M, Musolff A, Weiss H. Influences of meteorological parameters on indoor radon concentrations (222Rn) excluding the effects of forced ventilation and radon exhalation from soil and building materials. *Journal of environmental radioactivity* 2018; **192**: 81–5.
12. Washington JW, Rose AW. Regional and temporal relations of radon in soil gas to soil temperature and moisture. *Geophysical Research Letters* 1990; **17**(6): 829–32.
13. Apikyan S, Diamond D. Nuclear terrorism and national preparedness: Springer; 2015.
14. Liu M, Kang C-M, Wolfson JM, et al. Measurements of gross α-and β-activities of archived PM2. 5 and PM10 Teflon filter samples. *Environmental Science & Technology* 2020; **54**(19): 11780–8.
15. Li L, Blomberg AJ, Lawrence J, et al. A spatiotemporal ensemble model to predict gross beta particulate radioactivity across the contiguous United States. *Environ Int* 2021; **156**: 106643.
16. Environmental Law Institute. ELI's Database of State Indoor Air Quality Laws. https://www.eli.org/buildings/database-state-indoor-air-quality-laws (accessed Apr 23 2026).
17. Federal Reserve Bank of Atlanta. Home Ownership Affordability Monitor: HOAM County Data. https://www.atlantafed.org/research-and-data/data/home-ownership-affordability-monitor (accessed Apr 23 2026).
18. Center For International Earth Science Information Network-CIESIN-Columbia University. Gridded Population of the World, Version 4 (GPWv4): Population Density, Revision

11 (Version 4.11) In: NASA Socioeconomic Data and Applications Center (SEDAC), editor. Palisades, NY; 2017.
